# A chitosan-based cascade-responsive drug delivery system for triple-negative breast cancer therapy

**DOI:** 10.1186/s12951-019-0529-4

**Published:** 2019-09-10

**Authors:** Shiwei Niu, Gareth R. Williams, Jianrong Wu, Junzi Wu, Xuejing Zhang, Xia Chen, Shude Li, Jianlin Jiao, Li-Min Zhu

**Affiliations:** 1 College of Chemistry, Chemical Engineering and Biotechnology, Donghua University, Shanghai, 201620 People’s Republic of China; 2 UCL School of Pharmacy, University College London, 29-39 Brunswick Square, London, WC1N 1AX UK; 3 School of Basic Medicine, Yunnan University of Traditional Chinese Medicine, Kunming, 650500 People’s Republic of China; 4 Department of Biochemistry and Molecular Biology, School of Basic Medicine, Kunming Medical University, Kunming, 650500 People’s Republic of China; 5 Technology Transfer Center, Kunming Medical University, Kunming, 650031 China

**Keywords:** Chitosan, Doxorubicin, Triple-negative breast, Cascade responsive, Cell penetrating peptide

## Abstract

**Background:**

It is extremely difficult to develop targeted treatments for triple-negative breast (TNB) cancer, because these cells do not express any of the key biomarkers usually exploited for this goal.

**Results:**

In this work, we develop a solution in the form of a cascade responsive nanoplatform based on thermo-sensitive poly(*N*-vinylcaprolactam) (PNVCL)-chitosan (CS) nanoparticles (NPs). These are further modified with the cell penetrating peptide (CPP) and loaded with the chemotherapeutic drug doxorubicin (DOX). The base copolymer was optimized to undergo a phase change at the elevated temperatures of the tumor microenvironment. The acid-responsive properties of CS provide a second trigger for drug release, and the inclusion of CPP should ensure the formulations accumulate in cancerous tissue. The resultant CPP-CS-*co*-PNVCL NPs could self-assemble in aqueous media into spherical NPs of size < 200 nm and with low polydispersity. They are able to accommodate a high DOX loading (14.8% w/w). The NPs are found to be selectively taken up by cancerous cells both in vitro and in vivo, and result in less off-target cytotoxicity than treatment with DOX alone. In vivo experiments employing a TNB xenograft mouse model demonstrated a significant reduction in tumor volume and prolonging of life span, with no obvious systemic toxicity.

**Conclusions:**

The system developed in this work has the potential to provide new therapies for hard-to-treat cancers.

## Background

Despite many recent advances in the development of new therapeutics, cancer is still a leading cause of death worldwide [1]. More than 14 million new cancer cases are reported annually. One major challenge is in the treatment of breast cancer, which has a high recurrence rate (~ 40%) and accounts for more than 600,000 deaths every year [2]. Triple-negative breast (TNB) cancer, which is defined by a lack of expression of estrogen receptor (ER), progesterone receptor (PR), and human epidermal growth factor receptor-2 (HER2), has the poorest prognosis and the most limited therapeutic options [3]. This is because these receptors are typically used to target therapies to tumor cells, and thus it is very challenging to target TNB cancer cells [4, 5]. Systemic chemotherapy can be employed with TNB cancer [6], but since there is no targeting very significant damage is caused to healthy tissue [7]. A range of other limitations also hampers systemic therapies, including low bioavailability, rapid blood clearance, and poor drug solubility [8, 9].

To address these issues, nanoscale drug delivery systems (DDSs) have been widely explored for chemotherapy. There are four main advantages of such nanotechnology approaches: (1) polymeric nanocarriers can help solubilize hydrophobic drugs in aqueous media [10]; (2) nanocomposites with appropriate size (70–200 nm) have prolonged blood circulation times and can passively target tumor tissue through the enhanced permeability and retention (EPR) effect; (3) grafting a nanocarrier with stimuli-responsive materials endows the DDS with microenvironment-responsive properties which can be exploited to ensure drug release only at the target site; and, (4) the nanomaterials can be further functionalized with targeting ligands to achieve specific binding and uptake by tumor cells, thereby minimizing off-target side effects [11].

There has been great progress in nanoscience and nanotechnology over the past few decades, and a range of materials have been posited as novel approaches for cancer therapy. Among the various possible systems, self-assembled nanocarriers have attracted great interest as a result of their facile preparation process and ability to combine several features into one platform (e.g. protecting drugs from mononuclear phagocytosis and rapid clearance by the reticuloendothelial system [12]). Polymer nanoassemblies based on amphiphilic block copolymers which can self-assemble into “core–shell” structures in aqueous solution permit a hydrophobic drug cargo to be loaded into the core of the system, and thereby can enhance its water solubility [13, 14]. This is a major benefit given that many of the most potent chemotherapeutic active ingredients are poorly water soluble: their hydrophobicity is a major obstacle in ensuring they can circulate in the blood, for instance in the case of doxorubicin (DOX), an anthracycline anticancer drug approved by the Food and Drug Administration (FDA) which has extremely low water solubility [15, 16].

To make an effective DDS, the carrier itself must have high biocompatibility. The naturally-occurring biopolymer chitosan (CS) is one such material which has been widely explored. A range of CS-based self-assembling block copolymers and hydrogels have been constructed [17]. CS has good biocompatibility, biodegradability, and low immunogenicity [9, 18, 19]. Under the action of enzymes in vivo it degrades into the endogenous species water and carbon dioxide, ensuring no adverse effects from the degradation products [20]. CS is usually employed in the production of pH-sensitive DDSs, because of its enhanced solubility at slightly acidic pHs such as those found in the tumor microenvironment [21].

The tumor microenvironment is also characterized by hyperpyrexia (temperatures elevated to 40–42 °C because of the high glycolysis rate and rapid cellular proliferation [22]). Therefore, the development of materials which are thermo-responsive or dual-responsive to both temperature and pH could be a potent approach to improved targeting [23]. Thermo-responsive materials undergo a change in their physical properties driven by temperature. For instance, poly(*N*-vinylcaprolactam) (PNVCL) exhibits a reversible phase transition from hydrophilic to hydrophobic when the temperature is raised. This transition occurs at a temperature (termed the lower critical solution temperature, LCST) between 36 and 50 °C, depending on the polymer chain length [24]. The transition can be further tuned by incorporating co-monomers into the material [25, 26]. PNVCL is known to have excellent biocompatibility, stability to hydrolysis, and the ability to complex with other materials. Combined PNVCL-CS materials have attracted attention for use as dual pH/thermo-responsive antitumor DDSs [23, 26]. They can be further functionalized with active targeting moieties such as the cell penetrating peptide (CPP) [27]. Carcinomas are surrounded by a dense extracellular matrix full of matrix metalloproteinases (MMPs) and with a high interstitial fluid pressure (IFP) [28]. This makes it challenging for a DDS to penetrate the tumor, and CPP can be used to enhance transport across the stroma barriers [29–31].

**Scheme 1:**
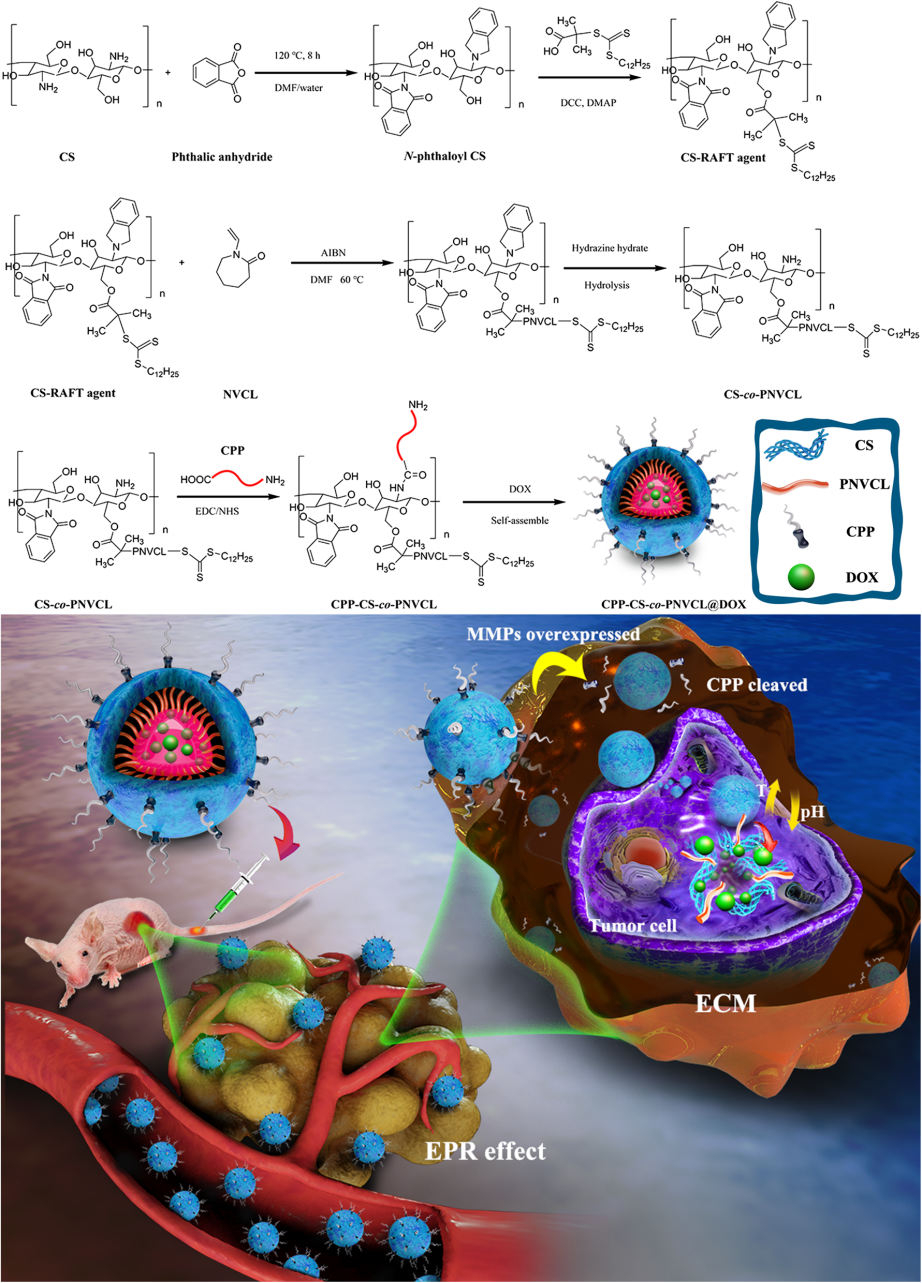
Schematic illustration of target-specific delivery of CPP-CS-*co*-PNVCL@DOX NPs to tumor cells

In this study, PNVCL was attached to a CS backbone through reversible addition fragmentation chain transfer (RAFT) polymerization. Subsequently, a tumor-homing CPP (RLYMRYYSPTTRRYG) was conjugated to CS-*co*-PNVCL through an amide bond. The resultant amphiphilic block copolymers should self-assemble into core/shell nanoparticles (NPs) in aqueous solution, allowing them to be loaded with a DOX cargo in the hydrophobic core to yield CPP-CS-*co*-PNVCL@DOX. The NPs are expected to remain intact in the systemic circulation owing to its neutral pH and the standard physiological temperature lying below the copolymer LCST [16]. As the NPs penetrate into the tumor tissue, the amide bond between CPP and CS will be cleaved by the MMPs in the ECM. The hydrophobic state of the polymer carrier at the elevated tumor temperature should enhance the interactions between the cell surface and NPs, extending the residence time in the tumor [23]. The mildly acidic conditions therein will cause the dissolution of CS, thereby triggering the release of the DOX inside the target cells. The strategy underlying this work is depicted in Scheme 1.

## Methods

### Materials and reagents

*N*-Vinylcaprolactam (NVCL) and anhydrous *N*,*N*′-dimethylformamide (DMF) were purchased from Sigma-Aldrich Co. (U.S.A.), and used as received. 2,2′-Azobis(2-methylpropionitrile) (AIBN) and *N*-hydroxysuccinimide (NHS) were obtained from GL Biochem (China). *N*,*N*-dicyclohexylcarbodimide (DCC) and *S*-1-dodecyl-*S*′-(α,α′-dimethyl-α″-acetic acid) trithiocarbonate (DDACT) were purchased from the Aladdin Company (China). Chitosan (CS, degree of deacetylation > 95%, molecular weight ca. 10 kDa), 4-(dimethyl-amino) pyridine (DMAP), phthalic anhydride, doxorubicin (DOX, > 98%) and 1-(3-dimethylaminopropyl)-3-ethylcarbodiimide hydrochloride (EDC·HCl) were acquired from J&K Scientific Ltd. (China). A tumor-lineage homing cell penetrating peptide (CPP, sequence RLYMRYYSPTTRRYG) was provided by Dangang Bio-Technology Co., Ltd. (China). Unless otherwise noted, all other materials were of analytical grade and used without further purification.

The human TNB breast cancer cell line MCF-7 was kindly provided by the American Type Culture Collection (ATCC, U.S.A.), and human umbilical vein endothelial (HUVEC) cells sourced from the Chinese Academy of Sciences (CAS, China). Adult Wistar rats (180–220 g) and female nude mice (16–22 g) were supplied by the Animal Center of Kunming Medical University (China) and housed under specific pathogen-free conditions with free access to standard food and water. Dulbecco’s Modified Eagle’s Medium (DMEM), fetal bovine serum (FBS), phosphate buffered saline (PBS), penicillin, streptomycin, and trypsin-ethylene diamine tetraacetic acid (trypsin–EDTA, 0.05%) were purchased from Corning (U.S.A.). Confocal dishes were obtained from Nunc (U.S.A.). 1,1-Dioctadecyl-3,3,3,3-tetramethylindotricarbocyanine iodide (DiR) and 4′,6-diamino-2-phenylindole (DAPI) were provided by Biotium (U.S.A.). MMP-2 and β-actin antibodies were purchased from Santa Cruz (U.S.A.), and an enhanced chemiluminescence (ECL) detection kit from Millipore Co., Ltd. (U.S.A.). A cell counting kit 8 (CCK-8) and Annexin V-FITC/PI cell apoptosis analysis kit were purchased from Beijing Solarbio Science & Technology (China). Terminal deoxynucleotidyl transferase-mediated dUTP nick-end labeling (TUNEL) and Ki67 detection kits, calcein-AM, propidium iodide (PI), and hematoxylin and eosin (H&E) were provided by Beyotime Biotechnology (China).

### Synthesis of CPP-CS-*co*-NVCL@DOX

The synthetic procedure is illustrated in Scheme 1. First, the CS backbone undergoes a full phthaloylation to protect the –NH_2_ functionality prior to further modifications. In brief, 10 mL of a phthalic anhydride solution (1.66 g of phthalic anhydride dissolved in 95/5 v/v DMF/water) was added to a CS solution (1 g CS in 100 mL of 1.0% v/v aqueous acetic acid), and the mixture heated at 120 °C under nitrogen with vigorous stirring for 8 h. The resultant mixture was cooled to room temperature and poured into ice water. The precipitate was collected by filtration, and then washed three times with 150 mL of methanol. This resulted in a pale tan powder (*N*-phthaloyl CS, 1.245 g, 55% yield). NVCL was then conjugated to CS by reversible addition fragmentation chain transfer (RAFT) polymerization. Following protocols described in a previous study [32], 0.36 g of *N*-phthaloyl CS was dispersed into 50 mL of dry DMF with ultrasonication, and DDACT (2.0 mmol), DCC (2.0 mmol) and DMAP (0.25 mmol) added. The mixture was stirred for 48 h at room temperature before being transferred into a dialysis bag (MWCO = 3500 Da) and dialyzed for 72 h against deionized water. Subsequently, the dialyzed product was lyophilized to yield a yellow powder (CS-RAFT agent, 0.415 g, 92% yield).

CS-*co*-PNVCL copolymers were synthesized at various monomer ratios to identify a polymer with an appropriate LCST. The CS-RAFT agent (0.05 g) was added into dry DMF (5 mL) and stirred magnetically under nitrogen until it had completely dissolved. Different mass ratios of PNVCL (0.5:1, 1:1, 2:1, 3:1 with respect to CS-RAFT) were added, and the reaction mixture heated to 60 °C in the presence of AIBN (0.0096 g, 0.06 mmol) under gentle stirring for 24 h. The reaction product was poured onto 30 mL of ice-cold diethyl ether, and the precipitate collected and dried under vacuum to obtain CS-*co*-PNVCL samples (termed CS-*co*-PNVCL_0.5_, CS-*co*-PNVCL_1_, CS-*co*-PNVCL_2_ and CS-*co*-PNVCL_3_ to reflect the mass ratios of PNVCL:CS-RAFT).

Subsequently, the NH_2_– groups of CS were deprotected by stirring in a hydrazine hydrate solution (1/99 v/v hydrazine hydrate/DMF, 30 mL) for 48 h at room temperature. 100 mg CPP was well dispersed in 5 mL deionized water, and a mixed EDC/NHS solution (52 mg of EDC, and 34 mg of NHS in 5 mL deionized water) was added for 2 h to activate its –COOH groups. Deprotected CS-*co*-PNVCL (0.50 g) was then added to give a ratio of –NH_2_:–COOH of 1:1. This mixed solution was reacted under gentle stirring at room temperature overnight. It was then subjected to a series of centrifugation steps (11,000 rpm, 10 min, 3 times) followed with washing with deionized water (10 mL) so as to remove any unreacted materials. Finally, the CPP-CS-*co*-PNVCL materials were recovered by freeze-drying. The average molecular weight (Mn) of the segments in the synthetic process was determined by gel permeation chromatography (GPC, Viscotek GPC270, Malvern, U.K.) using tetrahydrofuran (THF) as the eluent.

### LCST determination

The LCST behavior of the CPP-CS-*co*-PNVCL copolymers was probed using UV–Vis spectroscopy (UV-2450PC, Shimadzu, Japan). The transmittance of 0.25 wt% aqueous polymer solutions was measured at 500 nm in intervals of 2 °C over the range of 25–55 °C. The solution was equilibrated at each temperature for 20 min before transmittance was quantified [25].

### Drug loading

The CPP-CS-*co*-PNVCL_1_ copolymer was found to have the most appropriate LCST, and thus employed for all further experiments. An aqueous solution of this copolymer (0.50 g in 25 mL deionized water) was mixed with a DOX solution (0.10 g in 25 mL DMSO) and vigorously stirred at room temperature for 8 h. The drug/polymer mixture was then sealed in a dialysis bag (MWCO = 14,000 Da) and dialyzed against water for 72 h at room temperature, to yield CPP-CS-*co*-PNVCL_1_@DOX NPs.

To determine the drug loading (DL) and entrapment efficiency (EE) of DOX in CPP-CS-*co*-PNVCL_1_, the NPs were dissolved in DMSO and the concentration of DOX quantified at 481 nm. The DL and EE were calculated as:

$${\text{DL}}\left( \% \right) = {{\left( {{\text{Mass}}\;{\text{of}}\;{\text{DOX}}\;{\text{in}}\;{\text{NPs}}} \right)} \mathord{\left/ {\vphantom {{\left( {{\text{Mass}}\;{\text{of}}\;{\text{DOX}}\;{\text{in}}\;{\text{NPs}}} \right)} {\left( {{\text{Total}}\;{\text{mass}}\;{\text{of}}\;{\text{NPs}}} \right)}}} \right. \kern-0pt} {\left( {{\text{Total}}\;{\text{mass}}\;{\text{of}}\;{\text{NPs}}} \right)}} \times 100\%$$

$${\text{EE}}\left( \% \right) = {{\left( {{\text{Mass}}\;{\text{of}}\;{\text{DOX}}\;{\text{in}}\;{\text{NPs}}} \right)} \mathord{\left/ {\vphantom {{\left( {{\text{Mass}}\;{\text{of}}\;{\text{DOX}}\;{\text{in}}\;{\text{NPs}}} \right)} {\left( {{\text{Total}}\;{\text{mass}}\;{\text{of}}\;{\text{DOX}}} \right)}}} \right. \kern-0pt} {\left( {{\text{Total}}\;{\text{mass}}\;{\text{of}}\;{\text{DOX}}} \right)}} \times 100\% .$$

### Characterization

FT-IR spectra (range 4000–400 cm^−1^) were recorded on a Nicolet 6700 spectrometer (ThermoScientific, U.S.A.) by embedding the samples in KBr pellets. ^1^H NMR spectra were obtained using a Bruker AVANCE 400 M spectrometer (Bruker, U.S.A.) in D_2_O or DMSO-d_6_. UV–vis absorption spectra were quantified with a UV-2012 system (UNICO, China). The mean hydrodynamic size (PS), polydispersity index (PDI), and zeta potential (ZP) of the NPs were measured by dynamic light scattering (DLS) on a Zetasizer Nano ZS90 (Malvern, U.K.). Morphological examinations of the optimized CPP-CS-*co*-PNVCL_1_@DOX NPs were performed on a scanning electron microscope (SEM; Nova TM Nano instrument, FEI, U.S.A.). Samples for transmission electron microscopy (TEM) were prepared by placing a drop of NP suspension on a carbon-coated copper grid and leaving it to air dry. Images were obtained on a JEM-2100 instrument (JEOL, Japan).

### In vitro DOX release

To study the DOX release behavior in vitro, 5 mL of a 1 mg/mL dispersion of CPP-CS-*co*-PNVCL_1_@DOX NPs in PBS (pH 7.4 or pH 5.0) was transferred into a dialysis tube (MWCO = 10,000 Da) and dialyzed against 30 mL of the appropriate PBS solution. This process was performed at 25 °C, 37 °C or 40 °C, with shaking at 110 rpm. Aliquots of dialysate (1 mL) were collected at predetermined time intervals, and an equal volume of fresh pre-heated buffer added to maintain a constant volume. The concentration of DOX in the release medium was determined using UV–vis spectroscopy at 481 nm and the cumulative release calculated. The release experiments were carried out in triplicate, and the results reported as mean ± S.D.

### Cells and animals

Cells were cultured in DMEM supplemented with 10% (v/v) FBS and 1% (v/v) penicillin–streptomycin in a humidified atmosphere of 95% air and 5% CO_2_ at 37 °C. All animal care and handling was conducted in accordance with the Guide for the Care and Use of Laboratory Animals published by the US National Institutes of Health (NIH Publication No. 8523, revised 1985). All experimental protocols were approved by the Animal Care and Use Committee of Kunming Medical University (reference: KMMU 2015002).

### Cellular uptake in vitro

Cellular uptake was explored by confocal laser scanning microscopy (CLSM). Following a previously reported method [33], MCF-7 and HUVEC cells were seeded in confocal dishes. When the cells were 80% confluent, they were incubated with free DOX (dissolved in 1% v/v DMSO, 99% v/v PBS) or CPP-CS-*co*-PNVCL_1_@DOX NPs (dispersed in PBS) at a 50 μg/mL DOX concentration for 4 h at 37 °C. Additionally, a further sample of cells was incubated with the CPP-CS-*co*-PNVCL_1_@DOX NPs solution after preincubation with MMP-2 (2 mg/mL) for 1 h [34]. In all cases, after the 4 h incubation period the cells were washed with PBS (pH 7.4) three times, and fixed with a 2.5% v/v aqueous formaldehyde solution for 20 min, after which the nuclei were stained with DAPI (100 mg/mL) for 15 min. Cells were then again washed three times with PBS before being studied with CLSM (FV1000 microscope, Olympus, Japan).

The uptake profile was also investigated using flow cytometry. After incubation with free DOX or CPP-CS-*co*-PNVCL_1_@DOX NPs for 4 h, the cells were harvested, washed three times with PBS, and then resuspended in PBS. Flow cytometry was performed on an Accuri C6 instrument (BD, U.S.A.). 10,000 events were collected for each sample.

### Western blotting

MCF-7 and HUVEC cells were incubated as previously described for the cellular uptake assay, after which they were washed and lysed in modified RIPA buffer supplemented with 1:100 (v/v) of the proteinase/phosphatase inhibitor cocktail (Takara, China). After the quantification of total protein content, samples were subjected to sodium dodecyl sulfate polyacrylamide gel electrophoresis (SDS-PAGE) with a 10% gradient gel. The separated protein bands were transferred to PVDF membranes and then incubated with primary antibodies for β-actin (1:2000) and MMP-2 (1:500) overnight at 4 °C. After being washed three times with Tris-buffered saline containing 0.5% Tween-20, the gels were subjected to incubation at 37 °C with the appropriate HRP-conjugated secondary antibody (1:2000) for 2 h. Finally, the antigen–antibody complexes were visualized using an ECL detection kit. β-actin was employed as an internal reference, and relative expression quantified using ImageJ (National Institutes of Health, U.S.A.).

### Cell viability

For cytotoxicity evaluation, MCF-7 or HUVEV cells were seeded into a 96-well plate at 1 × 10^4^ cells/well (180 µL) and maintained at 37 °C. When the cells were 80% confluent, the medium was replaced with fresh medium containing the various formulations (DOX, CPP-CS-*co*-PNVCL_1_ and CPP-CS-*co*-PNVCL_1_@DOX) at DOX concentrations ranging from 0.001 to 10 μg/mL, and the cells incubated for 24 h in the dark. Cell viabilities were measured using a CCK-8 kit according to the manufacturer’s protocol, with the aid of a microplate reader (PowerWave XS, Bio-Tek, U.S.A.). Three independent experiments were performed with six replicate wells per experiment. Plots of cell viability versus the log of the drug concentration were used to estimate the half maximal inhibitory concentration (IC_50_) values from sigmoidal regressions.

Apoptotic cells were quantified using the Annexin V-FITC/PI double staining technique and quantified by flow cytometry. After treatment for 4 h with the different formulations (DOX, CPP-CS-*co*-PNVCL_1_ and CPP-CS-*co*-PNVCL_1_@DOX) at DOX concentration of 5 μg/mL, cells were trypsinized and collected, washed three times with PBS and then resuspended in PBS (1 × 10^6^ cells/mL). Thereafter, the suspensions were analysed using the Accuri C6 instrument to determine the proportions of cells that showed early apoptosis (positive for FITC-labeled Annexin V), late apoptosis (double positive for FITC-labeled Annexin V and propidium iodide), and necrosis (positive for propidium iodide).

To visualize cell apoptotisis, MCF-7 cells were seeded into six-well plates (2 mL, 5 × 10^5^ cells/well) and treated with the formulations (50 μg/mL DOX concentration, 200 µL/well) for 6 h. The medium was removed and the cells washed with PBS three times. Subsequently, a mixed Calcein-AM and PI solution was added for 15 min. The cells were then imaged on an inverted fluorescent microscope (TE-2000U, Nikon, Japan).

### Blood compatibility and circulation

To evaluate the pharmacological safety of the NPs, hemolysis assays was performed using rat red blood cells (RBCs). Fresh blood was collected from male Wistar rats (200–220 g), immediately transferred to heparinized tubes to prevent cruor, and centrifuged at 1500 rpm for 15 min to isolate RBCs. The RBCs were then resuspended in physiological saline at a 2% w/v concentration, followed by the addition of the formulations (DOX, CPP-CS-*co*-PNVCL_1_ and CPP-CS-*co*-PNVCL_1_@DOX). The mixture was incubated for 2 h at 37 °C in a thermostatic water bath. Subsequently, the RBCs were centrifuged for 15 min at 1500 rpm, and the supernatant collected. The amount of hemoglobin released was measured via a microplate reader (PowerWave XS, Bio-Tek, U.S.A.) at 540 nm. RBCs suspended in saline and 1% w/v Triton X-100 were used as controls. All experiments were performed in triplicate.

The pharmacokinetic profiles of DOX and CPP-CS-*co*-PNVCL_1_@DOX were probed in vivo by intravenous administration (5.0 mg DOX equiv. kg^−1^) into the tail vein of Wistar rats. At predetermined time points (0, 5, 30 min; 1, 2, 3, 4, 8, 12, 16, 20 and 24 h), blood (0.5 mL) was withdrawn from the ocular vein, and immediately transferred to heparinized tubes. The blood samples were centrifuged at 4000 rpm for 5 min, and the plasma collected. The concentration of DOX in the blood was then determined using UV/vis spectrophotometry.

### In vivo and ex vivo imaging

To monitor the metabolic fate of the NPs, a real-time in vivo imaging system was used to study the drug biodistribution at different timepoints in a xenograft model. The fluorescent probe DiR was encapsulated into the core of CPP-CS-*co*-PNVCL_1_ NPs following the same protocol as for DOX above (giving CPP-CS-*co*-PNVCL_1_@DiR NPs). A suspension of 5 × 10^6^ MCF-7 cells in DMEM (150 μL) was subcutaneously injected into the right buttock of BALB/c nude mouse (20–22 g) to establish a mammary carcinoma xenograft model. When the tumor volume reached approximately 150 mm^3^, free DiR (in 10 μL DMSO) or CPP-CS-*co*-PNVCL_1_@DiR NPs (in 10 μL saline) were injected *i.v.* into the mice at 7.5 mg DiR equiv. kg^−1^. At scheduled time points (1, 4, 10, and 24 h) postinjection, the mice were anaesthetized and imaged using a Maestro in vivo imaging system (CRi Inc., U.S.A.). Thereafter, the mice were sacrificed, and the heart, liver, spleen, lung, kidney, and tumor were excised to observe the DiR fluorescence distribution ex vivo.

### In vivo anticancer efficacy

Approximately 5 × 10^6^ MCF-7 cells (in 150 μL DMEM) were inoculated in the right buttock of nude mice. When the tumor size reached approximately 150 mm^3^, mice were randomly allocated into four groups (*n* = 10) and received one of the following treatments: (1) saline, (2) DOX, (3) CPP-CS-*co*-PNVCL_1_, (4) CPP-CS-*co*-PNVCL_1_@DOX. All the formulations were applied to the mice via *i.v.* injection at a dose of 7.5 mg/kg DOX every 2 days. Each time the mice were injected, the tumor size was measured using calipers and the volume calculated as (length × width^2^)/2. The weight of each mouse was also determined. The survival rate was evaluated by Kaplan–Meier analysis. After the course of treatment was finished, two mice from each group were anesthetized with phenobarbital (0.01 mg/kg), and blood samples collected via heart puncture. Thereafter, the mice were sacrificed, and the major organs (heart, lung, liver, spleen, and kidney) removed and fixed with 4% paraformaldehyde. Tumors were excised and divided into two parts, one of which was flash-frozen in liquid nitrogen and stored at − 80 °C while the second was fixed with 4% paraformaldehyde.

### Histopathological analysis

The excised organs were paraffin embedded and sectioned into 4 μm slices, then stained with H&E according to the manufacturer’s instructions. TUNEL assays and Ki67 immunohistochemistry were performed on tumor slices, with all protocols adhering to the manufacturers’ instructions. Photographs of the slices were obtained using an inverted microscope (C2 plus system, Nikon, Japan). Five microscopic fields of each slice were taken and further analyzed with the ImageJ software.

### Real-time reverse transcriptase polymerase chain reaction (RT-qPCR)

The total RNA of each stored tumor was isolated using the Trizol reagent (Promega, USA) according to the manufacturer’s instructions. 2 μg of total RNA from each sample was used for cDNA synthesis in a 25 μL reaction volume, following the vendor’s instructions (ThermoFisher, U.S.A.). 1 μL of each cDNA was used for was used for qRT-PCR analysis. Cycling conditions were as follows: 40 cycles of 94 °C for 1 min, 60 °C for 1 min, and 72 °C for 2 min. The fluorescence signal was determined at the end of each cycle, and the mRNA expression level of each target gene measured and normalized to GAPDH mRNA. The results were analyzed with the 2^−∆∆CT^ method. The primer sequences used are detailed in Additional file 1: Table S1.

### Biochemical index examinations

After blood samples were obtained, they were left to stand for 1 h prior to centrifugation for 20 min at 3500 rpm. The serum was then harvested. Liver function was assessed by measuring three well-known hepatic indicators [alanine aminotransferase (ALT), aspartate aminotransferase (AST), and alkaline phosphatase (ALP), and serum levels of urea nitrogen (BUN) and creatinine (CRE)] were determined to assess renal function. All these measurements were performed using an automated AU5400 biochemistry analyzer (Olympus, Japan).

### Statistical analysis

Statistical analysis was performed using the Student’s *T* test for comparison of two groups and one-way ANOVA for multiple groups, the latter followed by a Newman–Keuls test if the overall *P *< 0.05. A *P* value of less than 0.05 was considered significant (*), while a *P* value of less than 0.01 was considered highly significant (**).

## Results and discussion

### Phase transition behavior of CPP-CS-*co*-PNVCL copolymers

**Fig. 1:**
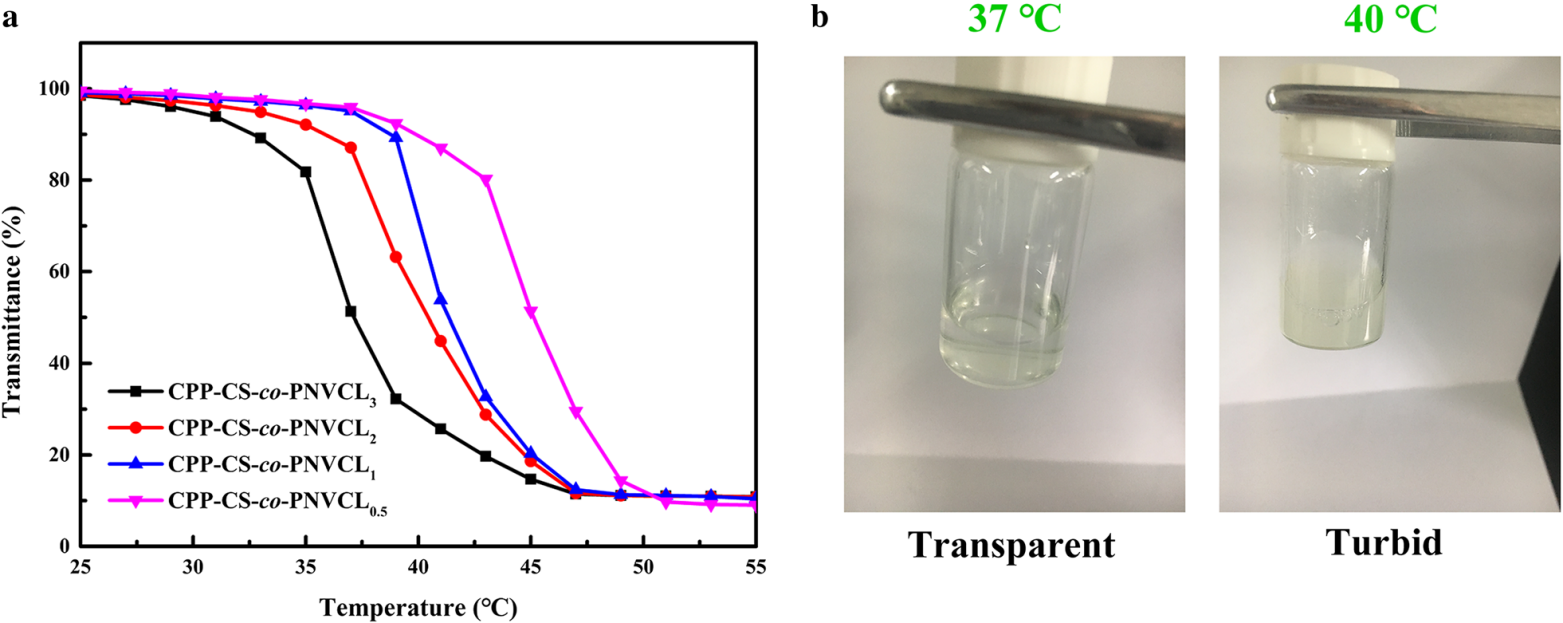
**a** UV transmittance at 500 nm of aqueous solutions of the CPP-CS-*co*-PNVCL copolymers as a function of temperature. **b** Photographs showing the change in optical transparency of the CPP-CS-*co*-PNVCL_1_ NPs at 37 °C (left) and 40 °C (right)

Since the phase transition temperature is a key parameter for targeted delivery to the tumor, the LCST of the CPP-CS-*co*-PNVCL materials in aqueous solution was first investigated. The CPP-CS-*co*-PNVCL copolymers with different ratios exhibited varied phase transition behavior, as shown in Fig. 1a. All of the copolymers showed a distinct phase change from hydrophilic to hydrophobic as the temperature was raised, with the lower critical solution temperature (LCST) decreasing with an increased content of PNVCL. For CPP-CS-*co*-PNVCL_1_, a LCST at around 40 °C (near tumor temperature) was clearly observed. Digital photographs (Fig. 1b) confirm the findings from UV spectroscopy. CPP-CS-*co*-PNVCL_1_ was transparent at the physiological temperature (37 °C) but became turbid once the temperature was raised to 40 °C. This phenomenon can be attributed to the disruption of hydrogen bonding with water and increasing hydrophobic interactions between the caprolactam groups at higher temperatures [26, 35]. Given it had the most appropriate LCST for the intended application, CPP-CS-*co*-PNVCL_1_ was employed in all subsequent experiments.

### Structural characterization of CPP-CS-*co*-PNVCL_1_@DOX

**Fig. 2:**
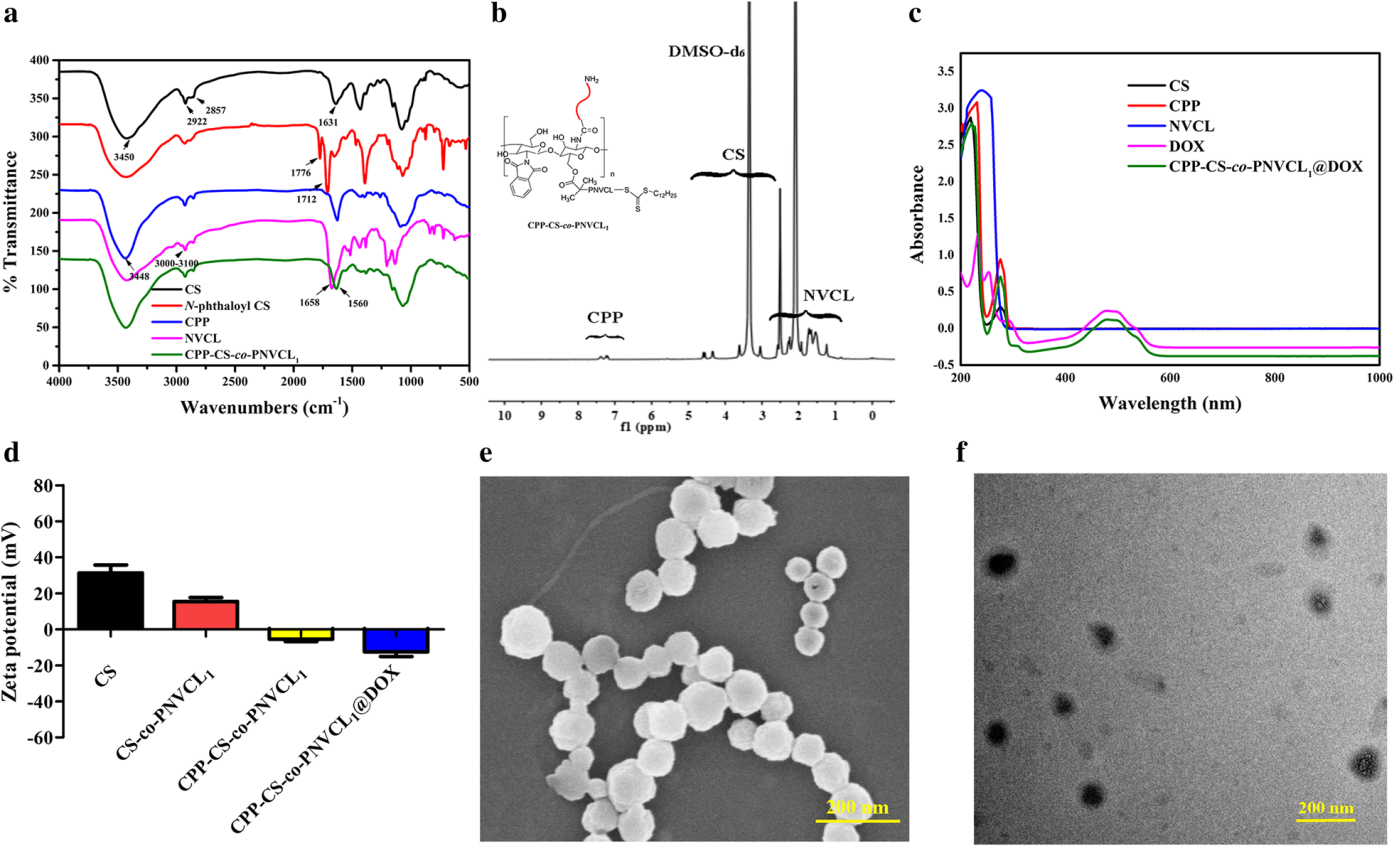
Structure and physicochemical characterization of the CPP-CS-*co*-PNVCL_1_@DOX NPs. **a** FT-IR spectra, **b** ^1^H NMR spectrum of the CPP-CS-*co*-PNVCL_1_@DOX NPs, **c** UV–vis spectra, **d** zeta potential values, with **e** SEM and **f** TEM images of the CPP-CS-*co*-PNVCL_1_@DOX NPs

FT-IR spectra of CS, *N*-phthaloyl CS, CPP, NVCL and the CPP-CS-*co*-PNVCL_1_ copolymer are shown in Fig. 2a. CS shows the amide I band at 1631 cm^−1^, C–N stretching at 2857 cm^−1^ and the characteristic stretching vibrations of hydroxyl, aliphatic C–H, and acetylated amino groups at 3450, 2922 and 1658 cm^−1^, respectively. Additional absorptions are noted at 1776 and 1712 cm^−1^ from imide groups in *N*-phthaloyl CS, which suggests the successful protection of the –NH_2_ groups on CS. The NVCL monomer displays features at 1658 cm^−1^ (C=C) and 3000–3100 cm^−1^ (CH= and CH_2_=). CPP has absorption bands at 3448 cm^−1^ which can be ascribed to the –COOH groups. The CPP-CS-*co*-PNVCL_1_ copolymer combines all the characteristic absorption bands of CS, CPP and NVCL, and the formation of amide bonds is confirmed by the observation of a vibration at 1560 cm^−1^. The C=C bond of NVCL is no longer visible after polymerisation. The spectra thus indicate the successful preparation of CPP-CS-*co*-PNVCL_1_.

The introduction of CPP and NVCL onto CS was confirmed by ^1^H NMR spectroscopy. The ^1^H NMR spectrum of CPP-CS-*co*-PNVCL_1_ (DMSO-d_6_) is given in Fig. 2b, and spectra from all the stages in the synthesis are provided in Additional file 1: Figure S1. Resonances from protons on the carbon backbone of CS be seen at 3.00–4.00 ppm (due to the electron absorption effect of carbonyl group after the formation of amide band, resulting in the chemical shift from the original CS), and a peak at 3.1 ppm is assigned to the proton of glucosamine residues. The characteristic resonances of NVCL are present at δ = 4.46 (2H, 4-H), 3.35 (2H, 1-H), 1.62 (2H, 6-H), 2.40 (2H, 5-H) and 1.14 (2H, 7-H. The peaks at 7.00–8.00 ppm are distinctive signals of the aromatic groups in CPP. The degree of substitution (DS) of the RAFT reagent and CPP were calculated to be ca. 21.7%, and 7.6% respectively, based on the ^1^H NMR spectrum. All these findings indicate the successful formation of CPP-CS-*co*-PNVCL_1_. The features observed are in good agreement with the literature [36]. The Mn data further support the stepwise construction of the polymer. After conjugation of the RAFT reagent to CS, the Mn increased from ca. 10 kDa (CS) to around 12.6 kDa (CS-RAFT). Addition of NVCL elevates the Mn further, with CS-*co*-PNVCL_1_ falling at approximately 13.8 kDa, and the final CPP-CS-*co*-PNVCL_1_ formulation has Mn ≈ 15.3 kDa.

DOX was loaded into the hydrophobic core of CPP-CS-*co*-PNVCL_1_ by self-assembly. UV–vis spectra of CS, CPP, NVCL, DOX and CPP-CS-*co*-PNVCL_1_@DOX NPs are depicted in Fig. 2c. The UV–vis spectrum of CPP-CS-*co*-PNVCL_1_@DOX clearly confirms the presence of CPP in the composite, because of the absorption peak at 290 nm. The absorption peak of DOX (481 nm) can also be seen in the spectrum of CPP-CS-*co*-PNVCL_1_@DOX, indicating that the drug had been successfully loaded into the NPs.

### Physicochemical characterization

The size and surface charge of the various NPs were monitored using DLS and zeta potential (ZP) measurements (Additional file 1: Figure S2A–D and Fig. 2d). All the NPs had a narrow particle size distribution, and the size increases going from CS (93 ± 10 nm) to CPP-CS-*co*-PNVCL_1_@DOX NPs (166 ± 19 nm). The CS NPs have a ZP of 31.3 ± 4.4 mV, due to the presence of –NH_2_ groups. The ZP declines to 15.4 ± 2.3 mV after formation of the CS-*co*-PNVCL_1_ copolymer, because the –NH_2_ groups of CS are protected by phthaloylation. Successful functionalization with CPP is confirmed by further changes in the ZP, which reduces again to − 5.5 ± 1.2 mV (attributed to the presence of carboxyl groups in CPP). After loading with DOX, the ZP reached its lowest value at − 12.5 ± 2.5 mV.

The size and size distribution of the NPs are suitable for intravenous administration in vivo, since the size of the NPs (below 200 nm) and narrow size distribution (PDI < 0.45) are appropriate for tumor targeting by the EPR effect [21, 37]. Moreover, the CPP-CS-*co*-PNVCL_1_@DOX NPs displayed a constant size upon storage as an aqueous suspension for 7 days, indicating good stability to aggregation (see Additional file 1: Figure S2E). Based on the these results, we expect that the CPP-CS-*co*-PNVCL_1_ NPs can avoid non-specific protein adsorption in the blood stream, resulting in prolonged circulation and permitting them accumulate in the tumor tissues via the EPR effect [38].

The morphology of the CPP-CS-*co*-PNVCL_1_@DOX NPs was visualized by both SEM and TEM (Fig. 2e, f). The images clearly revealed that CPP-CS-*co*-PNVCL_1_@DOX NPs have a relatively monodisperse size distribution with a mean diameter of ~ 120 ± 15 nm (*n* = 8). The NPs are spherical in shape. The size of the CPP-CS-*co*-PNVCL_1_@DOX NPs measured by TEM was slightly smaller than the DLS values, which is as expected and arises because the NPs are hydrated in aqueous media.

The DL of DOX was calculated to be 14.8 ± 1.8% and the EE 85.3 ± 9.7%. These values are higher than for previously reported CS-based NPs, which had a DL of 13.5% and EE of 74.3% [11], and indicate that the CPP-CS-*co*-PNVCL_1_@DOX NPs are suitable for the delivery of anticancer drugs.

### Stimuli-responsive behavior

**Fig. 3:**
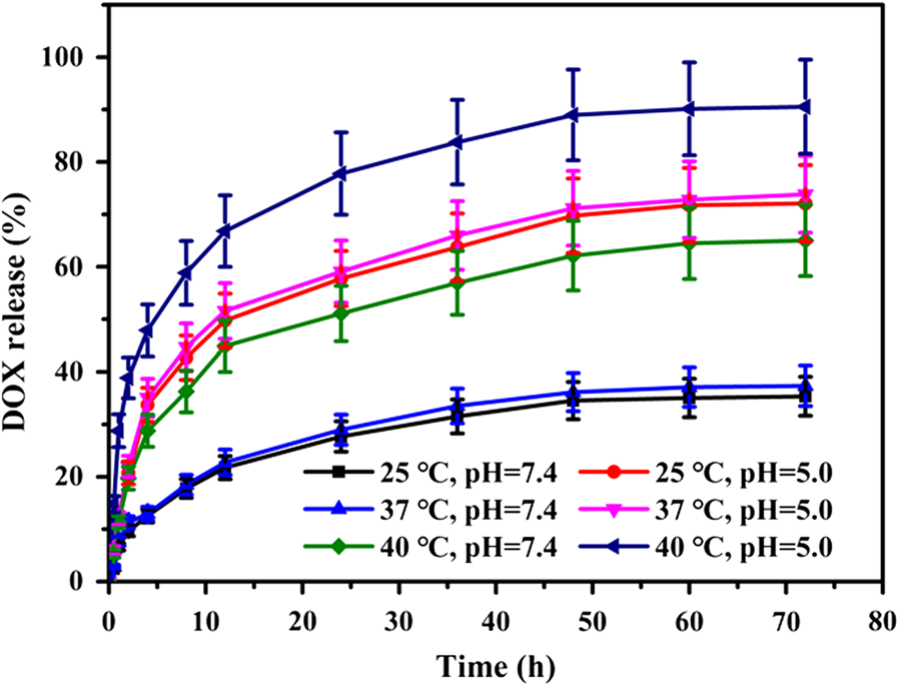
In vitro DOX release at different pHs and temperatures (*n* = 3, mean ± S.D)

The rate of DOX release varies with temperature and pH (Fig. 3) [39]. An initial burst of drug release was noted under all conditions, as a result of some drug being present at the surface of the NPs. After 24 h, ca. 28% of the DOX loading was released from the NPs at pH 7.4/25 °C, whereas 58% was released at pH 5.0/25 °C and 51% at pH 7.4/40 °C. The noticeably accelerated DOX release rate under the acidic and hyperpyrexia conditions typical of the tumor results from the phase transition of the PNVCL, and the solubility of the CS in acidic environments. Drug release at the physiological temperature of 37 °C was similar to that at 25 °C. The CPP-CS-*co*-PNVCL_1_@DOX NPs gave a greater amount of DOX release (91% after 72 h) at pH 5.0 and 40 °C. The latter is higher than other previously reported dual-responsive systems (less than 80% after 72 h) [12, 40]. The hybrid NPs clearly comprise an efficient temperature- and pH-responsive carrier system for tumor targeting.

### Cellular uptake

**Fig. 4:**
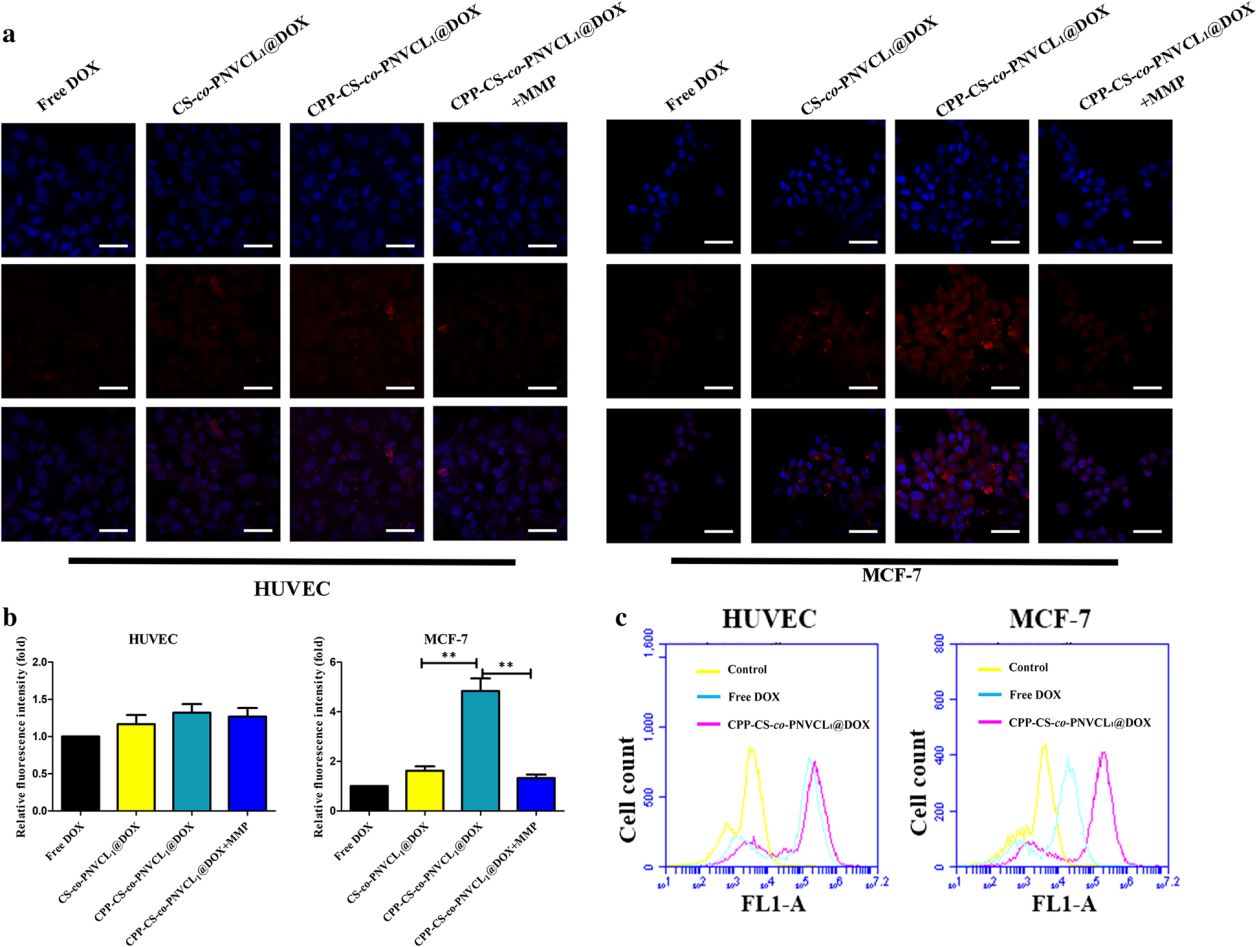
Cellular uptake data. **a** CLSM images of HUVEC and MCF-7 cells after incubation with free DOX, CS-*co*-PNVCL_1_@DOX, and CPP-CS-*co*-PNVCL_1_@DOX NPs with or without MMP-2 (equivalent DOX concentration: 50 μg/mL; scale bar = 50 μm). The DAPI-stained nuclei are blue, and DOX red. **b** A quantitative histogram profile of DOX cellular uptake. **c** The mean fluorescence intensity from DOX in HUVEC and MCF-7 cells after incubation with the different formulations, as determined using flow cytometry

Both healthy HUVEC and the triple-negative human breast cancer MCF-7 cells were used in these experiments. The enzyme MMP-2 is reported to be heavily secreted by the ECM into the pericellular space of tumor cells [33]. This was verified by western blotting analysis (Additional file 1: Figure S3), which shows that while the MCF-7 cells produce high levels of MMP-2 there is very little expression in HUVECs. The cellular uptake of the various formulations was analyzed by CLSM (Fig. 4a). In the case of the HUVEC cells, the DOX signals (red) were all very weak, regardless of whether the NPs had been pre-incubated with MMP or not [41]. Similarly, the MCF-7 cells exposed to free DOX or the CS-*co*-PNVCL_1_@DOX NPs showed low levels of uptake of DOX owing to the absence of any active cell entry mechanism. In contrast, the uptake of CPP-CS-*co*-PNVCL_1_@DOX NPs by MCF-7 cells was markedly increased. However, this uptake was reduced when the cells were preincubated with MMP-2. These results indicate that MMP-2 cleavage leads to the dissociation of CPP from the NPs, which removes the penetrating ability of the NPs. This means that when the NPs enter the tumor environment, it can be expected that the high levels of MMP-2 present will cleave the CPP from the particle surfaces, meaning they can no longer effectively be transferred through cell membranes and causing them to become localised in the tumor site [42].

A quantitative analysis of cellular uptake is depicted in Fig. 4b, and is in agreement with the microscopic observations. No significant differences between the treatment groups were seen with the HUVEC cells (*P* > 0.05). In contrast, MCF-7 cells treated with CPP-CS-*co*-PNVCL_1_@DOX NPs exhibited ca. 4.5-fold and 3.0-fold stronger red fluorescence than those exposed to free DOX and CS-*co*-PNVCL_1_@DOX NPs. When the CPP-CS-*co*-PNVCL_1_@DOX NPs were pre-incubated with MMP-2, the MCF-7 cells displayed equal red fluorescence to those which had been treated with free DOX. Flow cytometry data confirmed these findings (Fig. 4c). The NPs modified with CPP (CPP-CS-*co*-PNVCL_1_@DOX) could enhance the internalization of DOX in MCF-7 cells, but not in control HUVEC cells. The NPs thus have high specificity to cancer cells with elevated MMP-2 expression levels [43, 44]. The enhanced cellular uptake observed here with the CPP-CS-*co*-PNVCL_1_@DOX NPs is notably higher than for other targeted CS systems reported in the literature (these show 1.2- to 2.3-fold greater uptake in the target cell populations than in healthy cells) [45, 46], consistent with the significant targeting abilities of CPP.

### Cytotoxicity

**Fig. 5:**
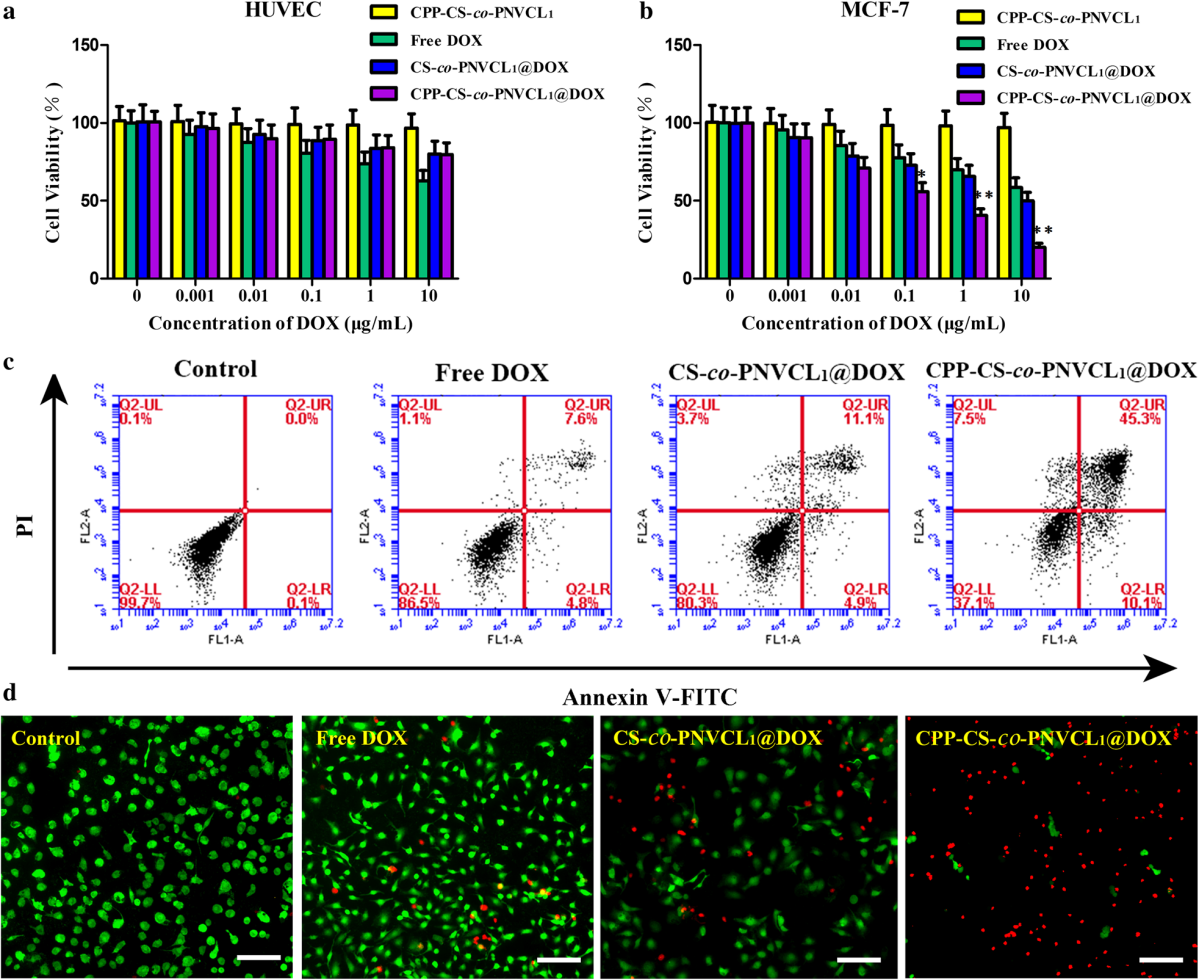
Cell viability of **a** HUVEC and **b** MCF-7 cells after incubation with CPP-CS-*co*-PNVCL_1_, free DOX, CS-*co*-PNVCL_1_@DOX and CPP-CS-*co*-PNVCL_1_@DOX NPs for 24 h. **c** Flow cytometry analysis of the death of MCF-7 cells. **d** Fluorescence images of calcein-AM (green)/PI (red) double stained cells treated with the different formulations (scale bars = 100 μm). Apoptotic cells show as having a red color

The cytocompatibility of drug-free CPP-CS-*co*-PNVCL_1_ NPs was next explored. As can be seen from Fig. 5a, b, the viabilities of both HUVEC and MCF-7 cell lines were > 90% even after incubation with the NPS at high concentrations (10 μg/mL), confirming the non-toxicity of the polymer material. Dose-dependent cytotoxicity was noted with free DOX (Fig. 5a, b). The CS-*co*-PNVCL_1_@DOX and CPP-CS-*co*-PNVCL_1_@DOX NPs both have similar cytotoxicity to HUVEC cells, with no significant differences between the two (*P* > 0.05). It is noticeable however that the NPs lead to less toxicity to these healthy cells than free DOX.

Significant differences between CS-*co*-PNVCL_1_@DOX and CPP-CS-*co*-PNVCL_1_@DOX NPs are seen against MCF-7 cells (Fig. 5b; *P* < 0.05). The cell viability decreased dramatically to ~ 20% when MCF-7 cells were exposed to CPP-CS-*co*-PNVCL_1_@DOX NPs for 24 h at a DOX dose of 10 μg/mL. This is expected to be due to the NPs accumulating in the tumor through CPP cleavage by MMP-2 enzymes over-expressed by the ECM of MCF-7, together with pH- and temperature-triggered DOX release [47]. Additional file 1: Figure S4 displays the IC_50_ values of the DOX formulations against MCF-7 cells; the value for the CPP-CS-*co*-PNVCL_1_@DOX NPs is 0.45 μg/mL, much lower than for DOX (13.5 μg/mL) and CS-*co*-PNVCL_1_@DOX (9.9 μg/mL).

The cell death profile of the formulations against MCF-7 cells was evaluated by flow cytometry and calcein-AM/PI double staining. Data from Annexin-V/propidiumiodide (PI) double staining are presented in Fig. 5c. The extent of cell death was calculated as the sum of the late apoptotic (upper left quadrant), early apoptotic (lower right quadrant) and necrotic (right upper quadrant) cells. The total percentage of dying cells with CPP-CS-*co*-PNVCL_1_@DOX is approximately 62.9%, much higher than with free DOX (13.5%) or CS-*co*-PNVCL_1_@DOX (19.7%). Calcein-AM/PI double staining (Fig. 5d) confirms these findings, revealing more apoptotic (red) MCF-7 cells after incubation with CPP-CS-*co*-PNVCL_1_@DOX NPs than with the other treatments.

### Hemocompatibility and pharmacokinetics

One of the major goals of this work was to reduce the inherent toxicity posed by DOX through its incorporation in a biocompatible vehicle. Hence, a hemolysis assay was performed to assess the NPs’ compatibility with blood (Additional file 1: Figure S5A). The degree of hemolysis induced by free DOX was proportional to its concentration, and severe RBC lysis was noted even at very low concentrations. In contrast, negligible hemolysis was observed for CPP-CS-*co*-PNVCL_1_-treated RBCs at concentrations below 250 μg/mL. CPP-CS-*co*-PNVCL_1_@DOX NPs caused some damage to RBCs, but the extent of hemolysis was dramatically lower than with free DOX. The CPP-CS-*co*-PNVCL_1_ NPs thus appear to have excellent hemocompatibility, indicating they are applicable for drug delivery in vivo.

The pharmacokinetics of free DOX and CPP-CS-*co*-PNVCL_1_@DOX NPs were studied in Wistar rats (Additional file 1: Figure S5B). The mean plasma DOX concentrations and area under the curve were notably increased when the CPP-CS-*co*-PNVCL_1_ NPs were used. The NPs showed a longer half life and were cleared much more slowly from the body than the pure drug. DOX from the CPP-CS-*co*-PNVCL_1_@DOX NPs can be detected in the blood for more than 20 h, and this prolonged circulation should lead to improved antitumor efficacy [48, 49].

### Biodistribution

**Fig. 6:**
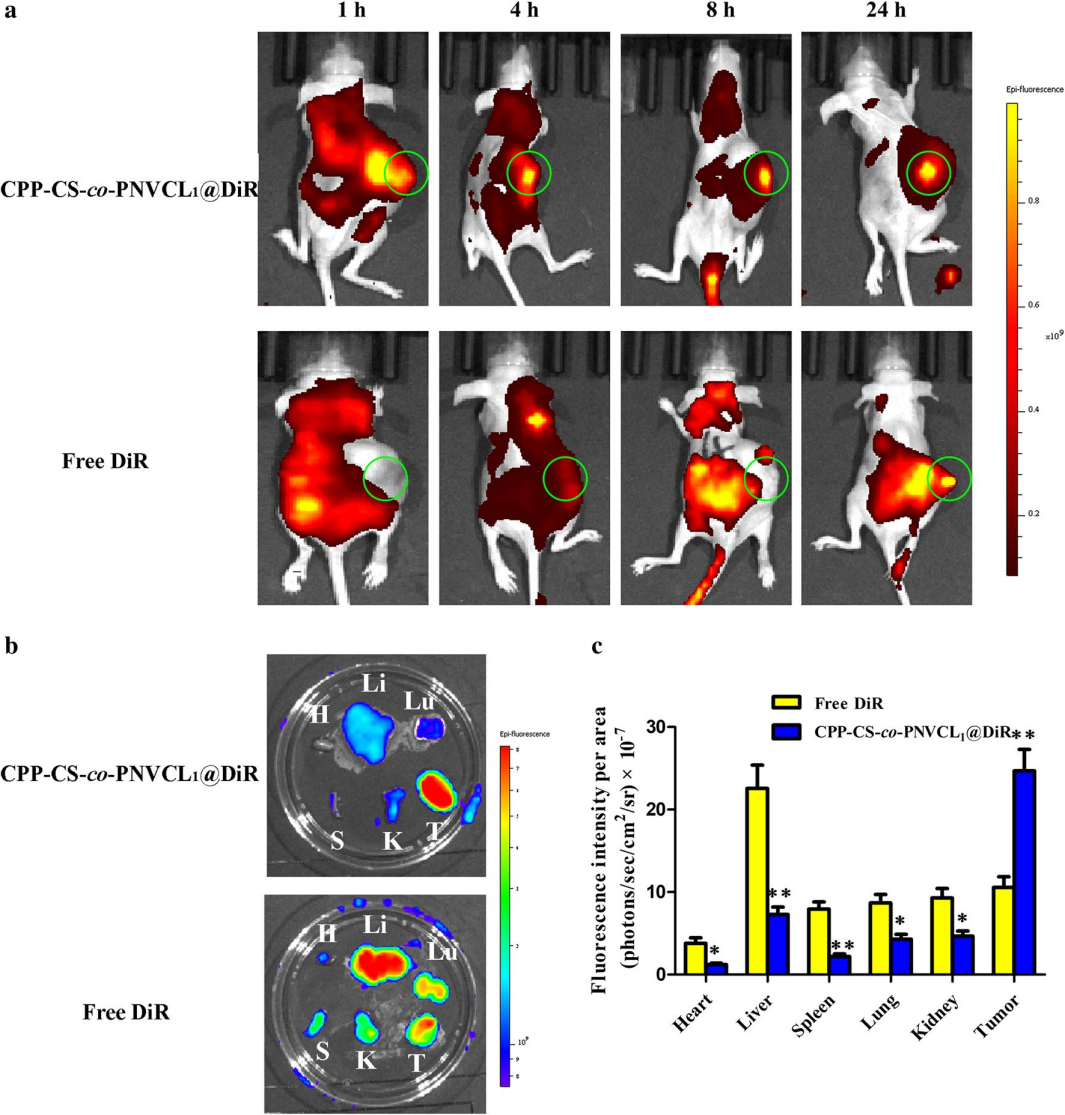
In vivo and ex vivo imaging data. **a** Real-time biodistribution of free DiR and CPP-CS-*co*-PNVCL_1_@DiR NPs after intravenous tail injection (circles indicate the tumor sites). **b** Ex vivo fluorescence images (H: heart, Li: liver, Lu: lung, S: spleen, K: kidney and T: tumor). **c** Ex vivo quantitative fluorescence intensity (*n *= 3; mean ± S.D, *P < 0.05, **P < 0.01 compared to free DiR)

Free DiR and DiR-labelled (CPP-CS-*co*-PNVCL_1_@DiR) NPs were injected into MCF-7 xenograft mouse models via *i.v.* tail injection, and in vivo images were captured at different time points post administration (see Fig. 6a). For the CPP-CS-*co*-PNVCL_1_@DiR NP group, high DiR fluorescence intensity was observed in the tumor even at 24 h post injection. This may be attributed to CPP-mediated targeting [50]. Free DiR exhibited much lower intra-tumor accumulation, and strong DiR fluorescence was observed in the liver and kidney. This is because DiR is hydrophobic, and thus is trafficked to these organs for onward metabolism [6]. When the mice were sacrificed, ex vivo fluorescence images (Fig. 6b) showed that CPP-CS-*co*-PNVCL_1_@DiR treated animals exhibited strong fluorescence signals in the tumor, while free DiR treated mice displayed a more uniform distribution of the fluorescence signal across the organs. Quantitative region of interest (ROI) analysis is shown in Fig. 6c. Compared to the free DiR group, the fluorescence signal of the CPP-CS-*co*-PNVCL_1_@DiR group was significantly increased in the tumor (*P* < 0.001), but significantly decreased in the heart, liver, spleen, lung and kidney (*P* < 0.05).

### In vivo anti-tumor activity

**Fig. 7:**
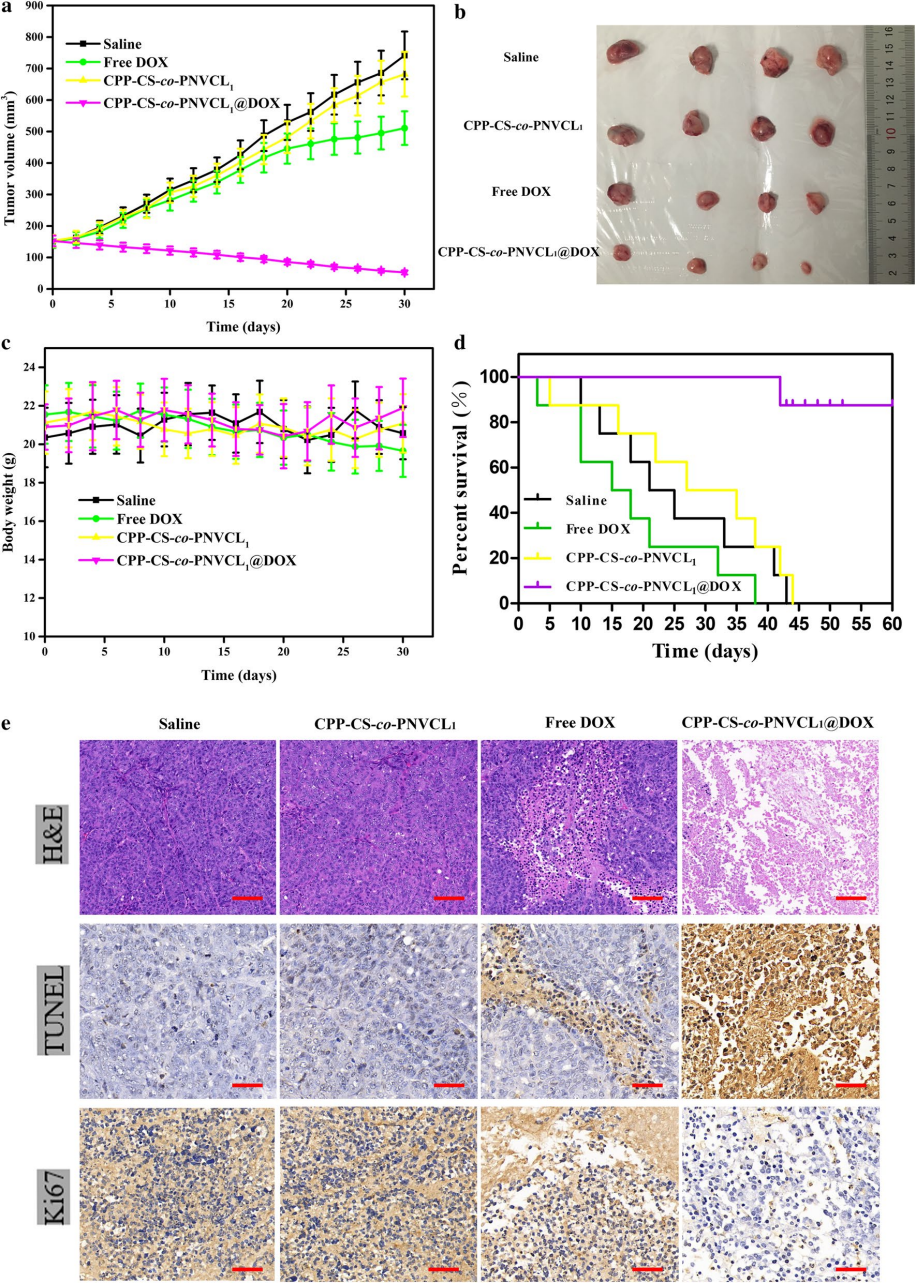
In vivo anticancer effects of free DOX and the NPs in MCF-7 xenograft nude mice. **a** Tumor volume changes; **b** digital photographs of the tumors after different treatments for 30 days; **c** body weight changes; **d** Kaplan–Meier survival curves (*n* = 8); **e** H&E, TUNEL and Ki67 staining assays performed on tumor tissues. Scale bars are 100 μm in the H&E assay and 50 μm in the TUNEL and Ki67 assays

MCF-7 tumor-bearing xenograft mice were devided into four groups and treated with saline (control), blank CPP-CS-*co*-PNVCL_1_ NPs, free DOX and CPP-CS-*co*-PNVCL_1_@DOX NPs. Tumor growth curves (Fig. 7a) reveal that tumors in the CPP-CS-*co*-PNVCL_1_ and saline groups both grew quickly. The free DOX group showed a slower tumor growth rate, while mice treated with CPP-CS-*co*-PNVCL_1_@DOX NPs exhibited a steady reduction in tumor volume. These findings are verified by digital photos of the mice taken after the experiment (Additional file 1: Figure S6) and of the excised tumors post-sacrifice (Fig. 7b). The body weight of the mice in all groups remained largely constant, except for the free DOX group where there is a modest decline (see Fig. 7c). This indicates there are no side-effects from the NPs.

The survival times of the xenograft mice were determined using the Kaplan–Meier method. The results (Fig. 7d) demonstrate that the mice receiving saline and CPP-CS-*co*-PNVCL_1_ had short lifespans due to rapid tumor growth. All the DOX treated mice were dead with 40 days, since although DOX has anti-tumor effects it also results in extensive systemic toxicity. The CPP-CS-*co*-PNVCL_1_@DOX group displayed the longest survival period, with more than 80% of the mice living for more than 60 days. These results offer an improvement over those previously reported in the literature (less than 60% of the treated mice living for more than 60 days) [9], and are supportive of the CPP-CS-*co*-PNVCL_1_@DOX NPs being more potent than similar temperature-responsive systems developed by others [18, 51].

The excised tumors were examined by H&E, Ki67 and TUNEL staining. Representative H&E images are depicted in Fig. 7e (upper panel). Negligible cell apoptosis was induced in the tumors of the saline and CPP-CS-*co*-PNVCL_1_ treatment groups. In contrast, the tumors treated with free DOX and CPP-CS-*co*-PNVCL_1_@DOX showed some degree of both necrosis and apoptosis, which is evident in looser tissue structures and signs of pyknosis and karyolysis. Mice treated with the CPP-CS-*co*-PNVCL_1_@DOX NPs displayed higher rates of cell death, in accordance with the anti-tumor results. The results of TUNEL (Fig. 7e, middle panel) and Ki67 (Fig. 7e, lower panel) show that tumors treated with CPP-CS-*co*-PNVCL_1_@DOX exhibited massive cell apoptosis, as well as the highest cell growth inhibition rate. This is evident in the maximum density of TUNEL-positive cells (apoptotic) and minimum density of Ki67-positive cells (those undergoing proliferation) with the CPP-CS-*co*-PNVCL_1_@DOX NPs. All these findings confirm the NPs to have extremely potent antitumor activity [52].

### Anti-tumor pro-apoptosis effects

In order to confirm the anti-tumor effects induced by CPP-CS-*co*-PNVCL_1_@DOX NPs, the apoptotic profile of tumoral cells was analysed on the molecular level. RT-qPCR was used to quantify the mRNA expression levels of key biomarkers [53, 54] including the proto-oncogene *Bcl*-*2*, pro-apoptosis factors (*Bax*, *Caspase*-*3*) and cancer suppressor genes (*PARP*, *PTEN* and *p53*). The results are shown in Additional file 1: Figure S7. No significant differences were found between the CPP-CS-*co*-PNVCL_1_ group and the saline control (*P *> 0.05), indicating the negligible cytotoxicity of the carrier. DOX treatment significantly (*P *< 0.05) suppressed the expression of *Bcl*-*2* mRNA, but the downregulation effect of the CPP-CS-*co*-PNVCL_1_@DOX NPs was more significant (*P *< 0.01). Both free DOX and CPP-CS-*co*-PNVCL_1_@DOX treatments upregulated the expression levels of *Bax*, *Caspase*-*3*, *PARP*, *PTEN* and *p53*. A significantly greater increase in the expression of these genes was observed in the CPP-CS-*co*-PNVCL_1_@DOX group (*P *< 0.01), suggesting the potential of the NPs to promote cancer cell apoptosis.

### Biosafety in vivo

**Fig. 8:**
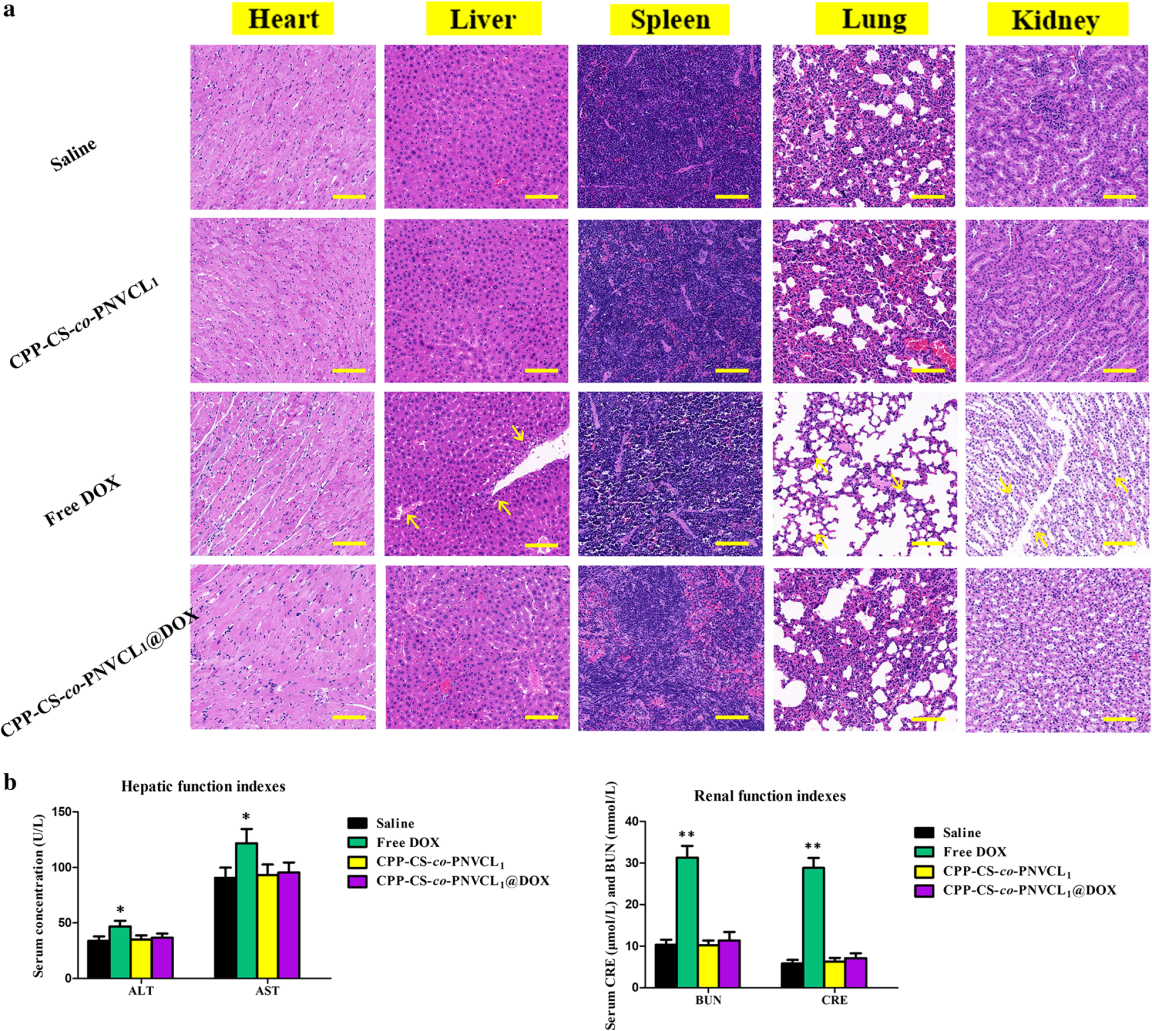
Biosafety data from in vivo studies after treatment for 30 days. **a** H&E analysis of the major organs (yellow arrows indicate cell necrosis, scale bars = 100 μm). **b** Concentrations of key biomarkers for side effects. Left histogram: serum hepatic function indices; right histogram: serum renal function indices. *P < 0.05, **P < 0.01 as compared to the saline group. Data are given as mean ± S.D (*n *= 6)

The toxicity of the various formulations was assessed by histological tissue imaging of the major organs (heart, liver, spleen, lung, and kidney) through H&E staining (Fig. 8a). Free DOX treatment led to vacuolar degeneration and necrosis in the liver, lung and kidney. In contrast, there were no evident pathological abnormalities in the various organs after exposure to CPP-CS-*co*-PNVCL_1_ and CPP-CS-*co*-PNVCL_1_@DOX; this confirms the lack of toxicity of the NPs to the major organs. Further, serum ALT/AST (liver function) and serum BUN/CRE levels (renal function) did not differ significantly between mice treated with saline, CPP-CS-*co*-PNVCL_1_ and CPP-CS-*co*-PNVCL_1_@DOX (Fig. 8b), indicating no liver or renal toxicity. In contrast, significant increases in all these markers were noted when the mice received free DOX treatment. Taken together, these data show that the CPP-CS-*co*-PNVCL_1_@DOX NPs are well tolerated and biocompatible. The biosafety of the NPs is in line with the best-performing biocompatible nanomaterials reported in the literature [55, 56].

## Conclusions

In this work, we have developed a novel responsive nanocarrier for the treatment of triple-negative breast cancer. This comprises chitosan (CS) copolymerized with *N*-vinylcaprolactam (NVCL) and functionalized with a cell penetrating peptide (CPP). The ratio of CS to NVCL was optimized to give a formulation which underwent a phase change at the elevated temperature of the tumor microenvironment, providing a route to targeted drug delivery. The inclusion of CS provides a second trigger, since it will dissolve selectively under acidic conditions. Finally, the presence of CPP will permit the system to accumulate in tumor cells. The resultant copolymer could be self-assembled into nanoparticles (NPs) in aqueous solution and loaded with the chemotherapeutic doxorubicin (DOX). We obtained high drug loading and encapsulation efficiency values (14.8 ± 1.8% and 85.3 ± 9.7% respectively), and observed drug release to be accelerated in acidic and hyperpyrexic conditions. The uptake of the NPs is elevated in human triple-negative MCF-7 cells, which leads to the selective death of cancer cells in vitro. In vivo experiments in a MCF-7 xenograft mouse model showed that the NPs accumulate at the tumor site after intravenous administration, and are highly effective in halting and reversing the growth of cancerous cells. The NPs have good hemocompatibility and biocompatibility in vivo, with no adverse off-target effects noted. Thus, the formulation developed is suitable for targeted triple negative breast cancer therapy. Moreover, since the design of this drug delivery system was undertaken considering the universal characteristics of the tumor microenvironment, the approach employed should be applicable to a variety of solid tumor types.

## Supplementary information

**Additional file 1: Table S1.** The primer sequences for *GAPDH*, *Bcl-2*, *Bax*, *Caspase-3*, *PARP*, *PTEN* and *p53*. **Figure S1.** ^1^H NMR spectra of (A) CS in D_2_O, (B) CS-RAFT in DMSO-d_6_ and (C) CS-*co*-PNVCL_1_ in DMSO-d_6_. **Figure S2.** Size distributions of NPs formed from (A) CS, (B) CS-*co*-PNVCL, (C) CPP-CS-*co*-PNVCL_1_, and (D) CPP-CS-*co*-PNVCL_1_@DOX NPs. (E) The particle size of the CPP-CS-*co*-PNVCL_1_@DOX NPs during storage for 7 days. Values are given as mean ± S.D (*n* = 3). **Figure S3.** MMP-2 protein expression levels in HUVEC and MCF-7 cells. **P < 0.01. Data are given as mean ± S.D (*n* = 3). **Figure S4.** IC_50_ values against MCF-7 cells for the different formulations (*n* = 6; results are shown as mean ± S.D, *P < 0.05, **P < 0.01 compared to free DOX). **Figure S5.** Hemocompatibility and pharmacokinetics. (A) Hemolytic activity of free DOX, CPP-CS-*co*-PNVCL_1_ and CPP-CS-*co*-PNVCL_1_@DOX NPs on rat red blood cells (*n* = 3, mean ± S.D). (B) Plasma concentration versus time curves for free DOX and CPP-CS-*co*-PNVCL_1_@DOX NPs in SD rats (*n* = 3, mean ± S.D). **Figure S6.** Representative images of tumors in MCF-7 xenograft nude mice after treatment for 30 days. The arrows indicate the tumor foci. **Figure S7.** mRNA expression levels for *Bcl-2*, *Bax*, *Caspase-3*, *PARP*, *PTEN*, and *p53* in the tumor tissues of MCF-7 tumor-bearing mice after treatment for 30 days. *n* = 6, results shown as mean ± S.D; *P < 0.05, **P < 0.01 as compared to the saline group.

## Data Availability

All data generated or analysed during this study are included in this published article.
