# Supplementary material for: A chitosan-based cascade-responsive drug delivery system for triple-negative breast cancer therapy
Source: J Nanobiotechnology. 2019 Sep 10;17:95. doi: 10.1186/s12951-019-0529-4 (PMC6737697; doi:10.1186/s12951-019-0529-4)
Supplement: Supplementary file 1 — Additional file 1: Table S1. The primer sequences for GAPDH, Bcl-2, Bax, Caspase-3, PARP, PTEN and p53. Figure S1. 1H NMR spectra of (A) CS in D2O, (B) CS-RAFT in DMSO-d6 and (C) CS-co-PNVCL1 in DMSO-d6. Figure S2. Size distributions of NPs formed from (A) CS, (B) CS-co-PNVCL, (C) CPP-CS-co-PNVCL1, and (D) CPP-CS-co-PNVCL1@DOX NPs. (E) The particle size of the CPP-CS-co-PNVCL1@DOX NPs during storage for 7 days. Values are given as mean ± S.D (n = 3). Figure S3. MMP-2 protein expression levels in HUVEC and MCF-7 cells. **P < 0.01. Data are given as mean ± S.D (n = 3). Figure S4. IC50 values against MCF-7 cells for the different formulations (n = 6; results are shown as mean ± S.D, *P < 0.05, **P < 0.01 compared to free DOX). Figure S5. Hemocompatibility and pharmacokinetics. (A) Hemolytic activity of free DOX, CPP-CS-co-PNVCL1 and CPP-CS-co-PNVCL1@DOX NPs on rat red blood cells (n = 3, mean ± S.D). (B) Plasma concentration versus time curves for free DOX and CPP-CS-co-PNVCL1@DOX NPs in SD rats (n = 3, mean ± S.D). Figure S6. Representative images of tumors in MCF-7 xenograft nude mice after treatment for 30 days. The arrows indicate the tumor foci. Figure S7. mRNA expression levels for Bcl-2, Bax, Caspase-3, PARP, PTEN, and p53 in the tumor tissues of MCF-7 tumor-bearing mice after treatment for 30 days. n = 6, results shown as mean ± S.D; *P < 0.05, **P < 0.01 as compared to the saline group. [file 12951_2019_529_MOESM1_ESM.docx]

**Additional file 1**

**A chitosan-based cascade-responsive drug delivery system for** **triple-negative breast cancer therapy**

Shiwei Niu^1^, Gareth R. Williams^2^, Jianrong Wu^1^, Junzi Wu^3^, Xuejing Zhang^1^, Xia Chen^1^, Shude Li^4^, Jianlin Jiao^5*^and Li-Min Zhu^1*^

^1^College of Chemistry, Chemical Engineering and Biotechnology, Donghua University, Shanghai, 201620, PR China

^2^UCL School of Pharmacy, University College London, 29-39 Brunswick Square, London WC1N 1AX, UK

^3^School of Basic Medicine, Yunnan University of Traditional Chinese Medicine, Kunming 650500, PR China

^4^Department of Biochemistry and Molecular Biology, School of Basic Medicine, Kunming Medical University, Kunming 650500, PR China

^5^Technology Transfer Center, Kunming Medical University, Kunming, 650031, China

Correspondence: [lzhu@dhu.edu.cn](mailto:lzhu@dhu.edu.cn); [jiaojianlin66@163.com](mailto:jiaojianlin66@163.com)

**Table S1.** The primer sequences for *GAPDH*, *Bcl-2*, *Bax*, *Caspase-3*, *PARP*, *PTEN* and *p53*.

| **Primer** | **Direction** | **Sequence** |
| --- | --- | --- |
| *GAPDH* | Forward | AAGAGGGATGCTGCCCTTAC |
|  | Reverse | ATCCGTTCACACCGACCTTC |
| *Bcl-2* | Forward | GACTGAGTACCTGAACCGGC |
|  | Reverse | TCACTTGTGGCCCAGGTATG |
| *Bax* | Forward | CTGGATCCAAGACCAGGGTG |
|  | Reverse | GTGAGGACTCCAGCCACAAA |
| *Caspase-3* | Forward | AGCTTGGAACGGTACGCTAA |
|  | Reverse | CCACTGACTTGCTCCCATGT |
| *PARP* | Forward | GCGGAGAAGACATTGGGTGA |
|  | Reverse | ACCATCTTCTTGGACAGGCG |
| *PTEN* | Forward | TTGTTAGCCTCTTGATGTGTGC |
|  | Reverse | CCATTGGTAGCCAAACGGAA |
| *p53* | Forward | AAACGCTTCGAGATGTTCCG |
|  | Reverse | CAAGGCTTGGAAGGCTCTAGG |


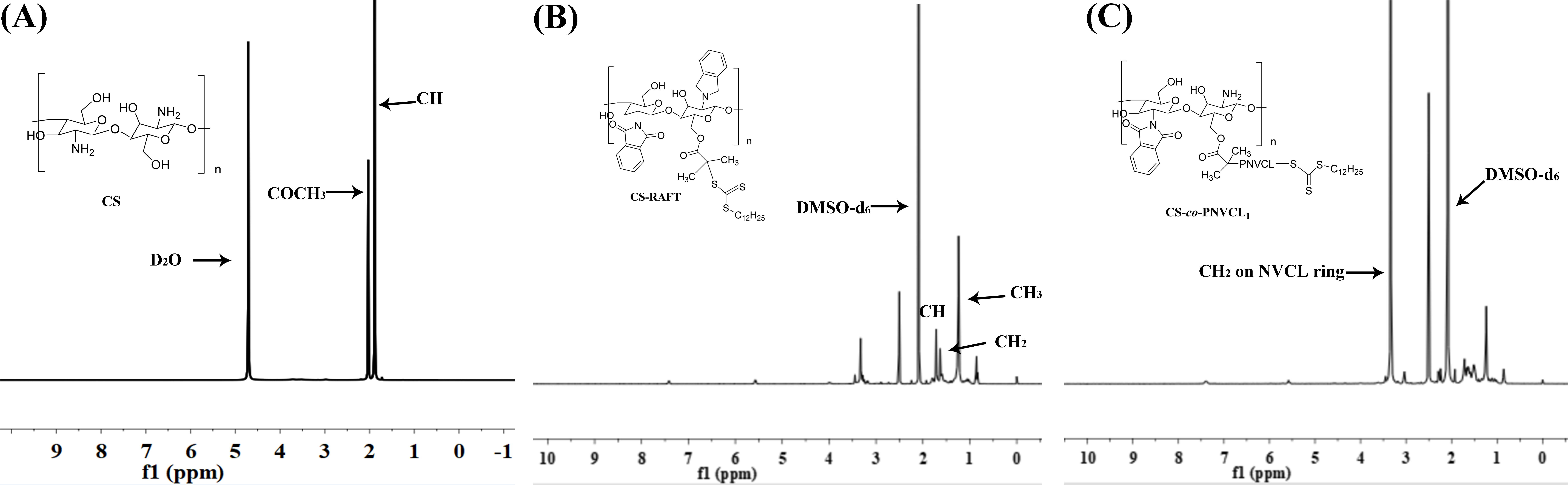


**Fig. S1** ^1^H NMR spectra of (A) CS in D_2_O, (B) CS-RAFT in DMSO-d_6_ and (C) CS-*co*-PNVCL_1_ in DMSO-d_6_.





**Fig. S2** Size distributions of NPs formed from (A) CS, (B) CS-*co*-PNVCL, (C) CPP-CS-*co*-PNVCL_1_, and (D) CPP-CS-*co*-PNVCL_1_@DOX NPs. (E) The particle size of the CPP-CS-*co*-PNVCL_1_@DOX NPs during storage for 7 days. Values are given as mean ± S.D. (*n* = 3).


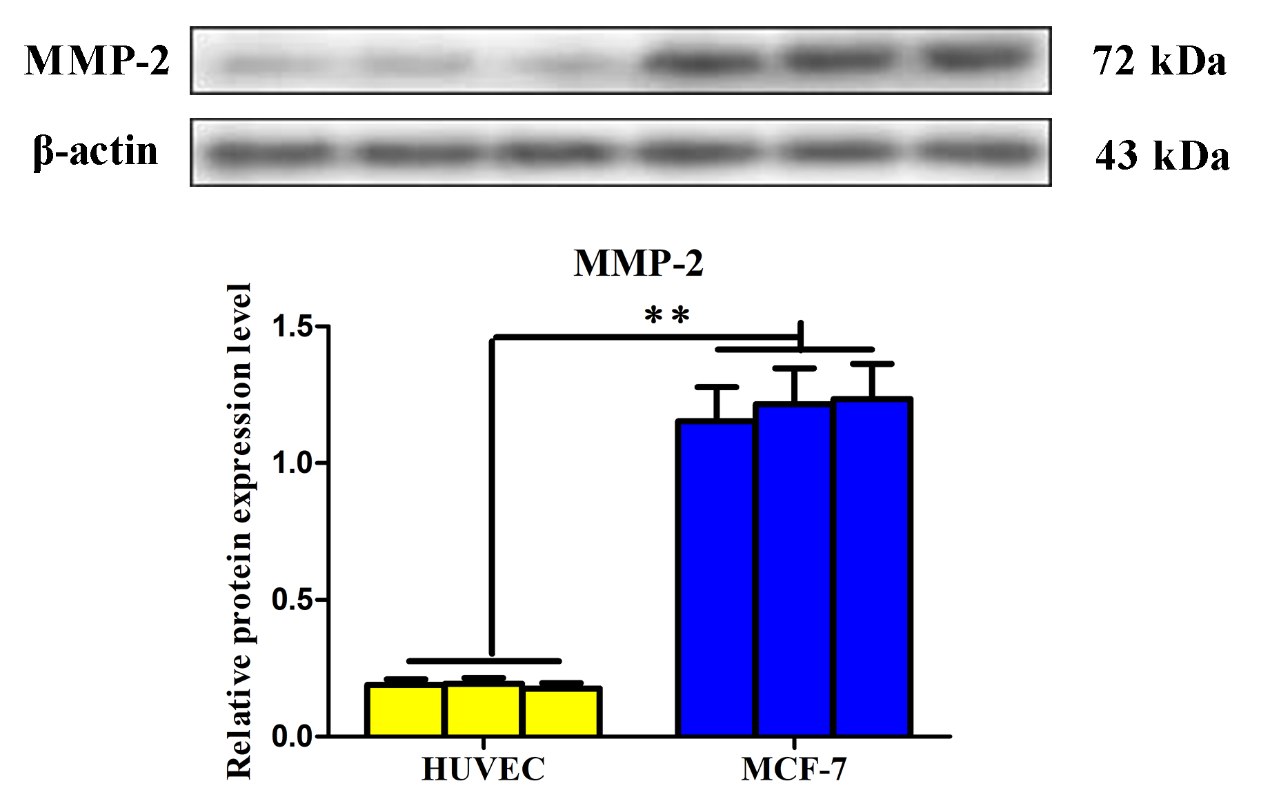


**Fig. S3** MMP-2 protein expression levels in HUVEC and MCF-7 cells. ***P* < 0.01. Data are given as mean ± S.D. (*n* = 3).


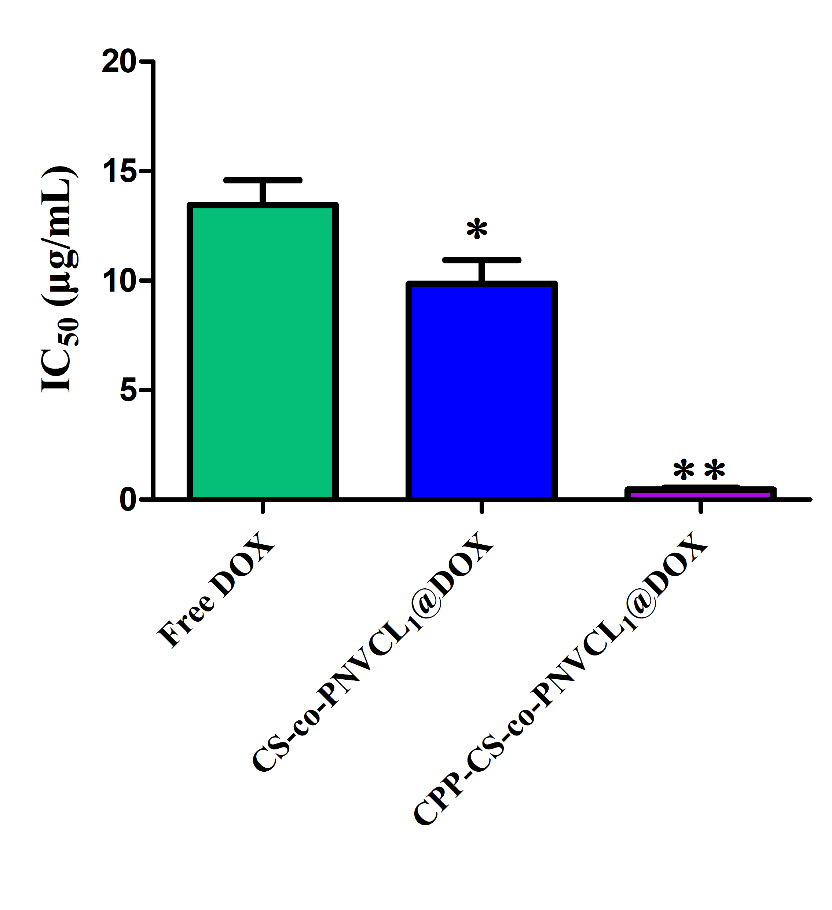


**Fig. S4** IC_50_ values against MCF-7 cells for the different formulations (*n* =6; results are shown as mean ± S.D., * *P* < 0.05, ** *P* < 0.01 compared to free DOX).


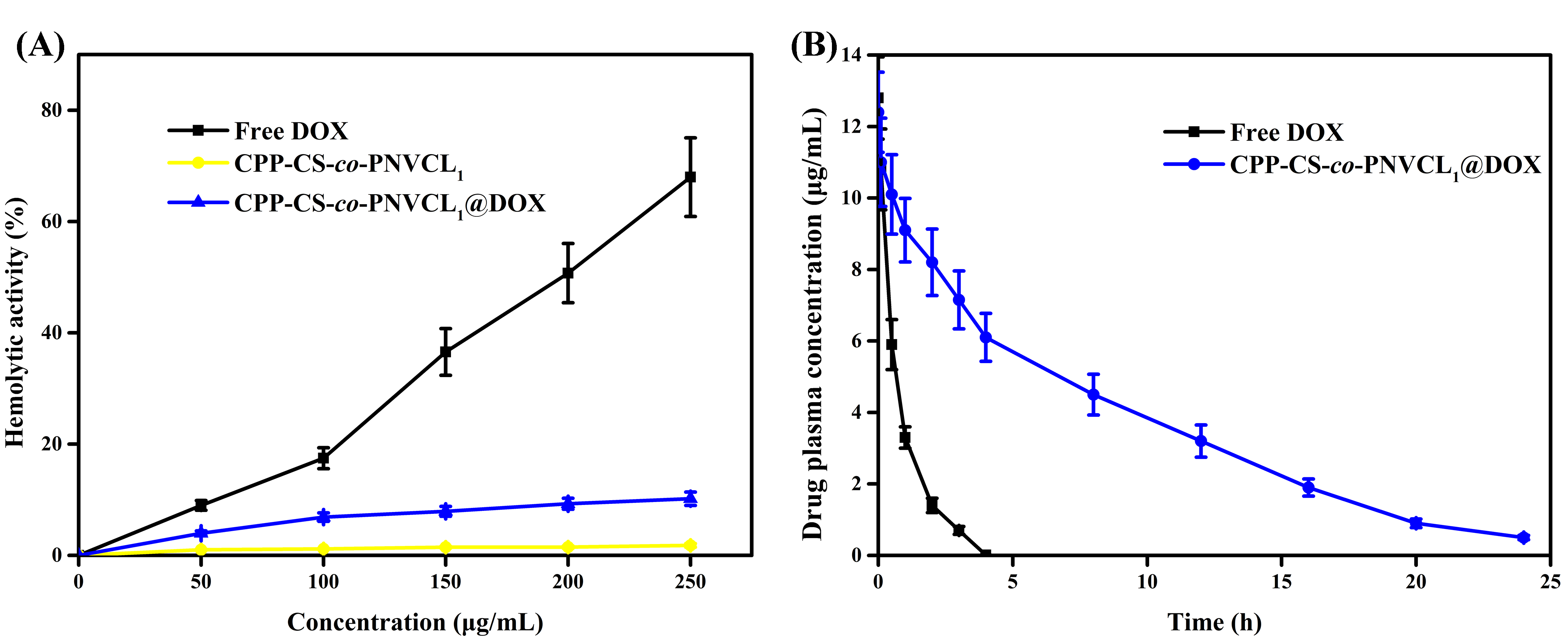


**Fig. S5** Hemocompatibility and pharmacokinetics. (A) Hemolytic activity of free DOX, CPP-CS-*co*-PNVCL_1_ and CPP-CS-*co*-PNVCL_1_@DOX NPs on rat red blood cells (*n* = 3, mean ± S.D.). (B) Plasma concentration versus time curves for free DOX and CPP-CS-*co*-PNVCL_1_@DOX NPs in SD rats (*n* = 3, mean ± S.D.).


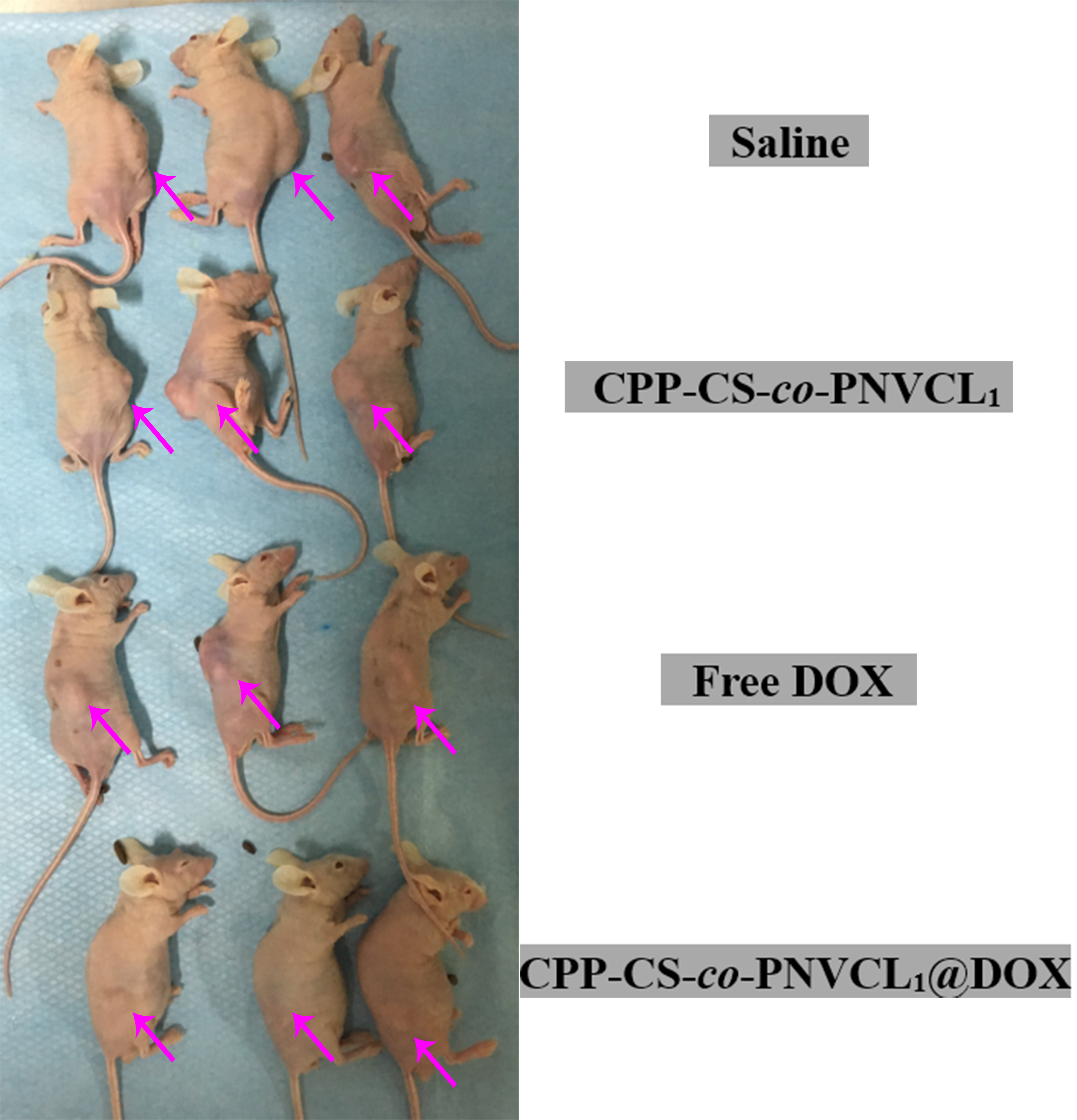


**Fig. S6** Representative images of tumors in MCF-7 xenograft nude mice after treatment for 30 days. The arrows indicate the tumor foci.


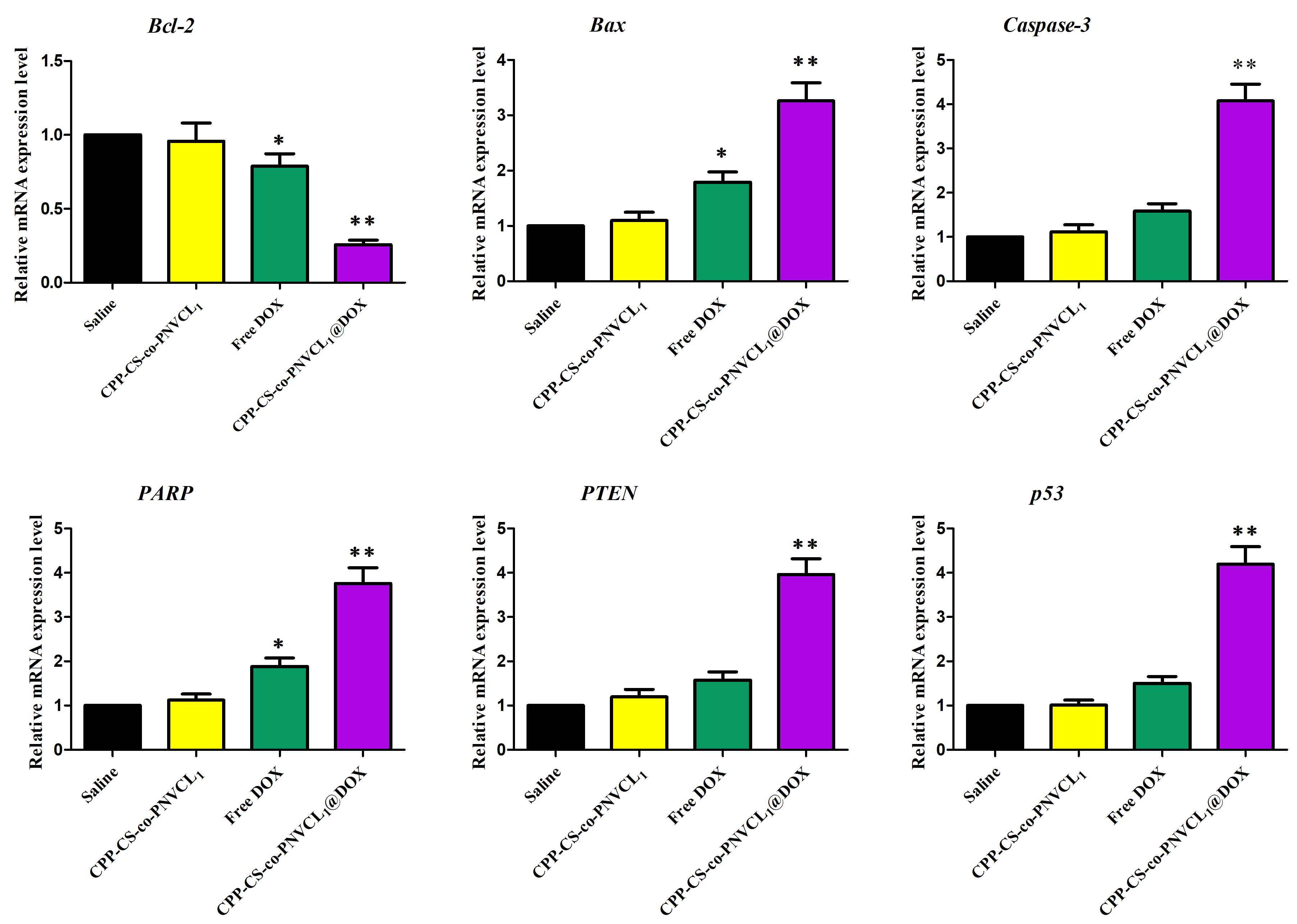


**Fig. S7** mRNA expression levels for *Bcl-2*, *Bax*, *Caspase-3*, *PARP*, *PTEN*, and *p53* in the tumor tissues of MCF-7 tumor-bearing mice after treatment for 30 days. *n* = 6, results shown as mean ± S.D.; * *P* < 0.05, ** *P* < 0.01 as compared to the saline group.
